# Neutrophils incite and macrophages avert electrical storm after myocardial infarction

**DOI:** 10.1038/s44161-022-00094-w

**Published:** 2022-07-11

**Authors:** Jana Grune, Andrew J. M. Lewis, Masahiro Yamazoe, Maarten Hulsmans, David Rohde, Ling Xiao, Shuang Zhang, Christiane Ott, David M. Calcagno, Yirong Zhou, Kerstin Timm, Mayooran Shanmuganathan, Fadi E. Pulous, Maximilian J. Schloss, Brody H. Foy, Diane Capen, Claudio Vinegoni, Gregory R. Wojtkiewicz, Yoshiko Iwamoto, Tilman Grune, Dennis Brown, John Higgins, Vanessa M. Ferreira, Neil Herring, Keith M. Channon, Stefan Neubauer, Oxford Acute Myocardial Infarction (OxAMI) Study, David E. Sosnovik, David J. Milan, Filip K. Swirski, Kevin R. King, Aaron D. Aguirre, Patrick T. Ellinor, Matthias Nahrendorf

**Affiliations:** 1 Center for Systems Biology, Massachusetts General Hospital and Harvard Medical School, Boston, MA, USA; 2 Department of Radiology, Massachusetts General Hospital and Harvard Medical School, Boston, MA, USA; 3 Cardiovascular Research Center, Massachusetts General Hospital and Harvard Medical School, Boston, MA, USA; 4 DZHK (German Centre for Cardiovascular Research), Partner Site Berlin, Berlin, Germany; 5 Department of Molecular Toxicology, German Institute of Human Nutrition Potsdam-Rehbruecke (DIfE), Nuthetal, Germany; 6 Department of Bioengineering, University of California, San Diego, La Jolla, CA, USA; 7 Wellman Center for Photomedicine, Massachusetts General Hospital and Harvard Medical School, Boston, MA, USA; 8 Department of Pharmacology, University of Oxford, Oxford, UK; 9 Radcliffe Department of Medicine, University of Oxford, Oxford, UK; 10 National Institute for Health (NIHR) Biomedical Research Centre, Oxford University Hospitals NHS Foundation Trust, John Radcliffe Hospital, Oxford, UK; 11 Department of Pathology, Massachusetts General Hospital, Boston, MA, USA; 12 Program in Membrane Biology, Nephrology Division, Department of Medicine, Massachusetts General Hospital and Harvard Medical School, Boston, MA, USA; 13 Department of Physiology, Anatomy and Genetics, University of Oxford, Oxford, UK; 14 Martinos Center for Biomedical Imaging, Massachusetts General Hospital and Harvard Medical School, Boston, MA, USA; 15 Division of Cardiology, Massachusetts General Hospital, Harvard Medical School, Boston, MA, USA; 16 Leducq Foundation, Boston, MA, USA; 17 Cardiovascular Research Institute and Department of Medicine, Icahn School of Medicine at Mount Sinai, New York, NY, USA; 18 Department of Medicine, Division of Cardiovascular Medicine, University of California, San Diego La Jolla, CA, USA; 19 The Broad Institute of MIT and Harvard, Cambridge, MA, USA; 20 Department of Internal Medicine, University Hospital Wuerzburg, Wuerzburg, Germany; 23 These authors contributed equally and are listed in alphabetical order: Andrew J. M. Lewis, Masahiro Yamazoe

## Abstract

Sudden cardiac death, arising from abnormal electrical conduction, occurs frequently in patients with coronary heart disease. Myocardial ischemia simultaneously induces arrhythmia and massive myocardial leukocyte changes. In this study, we optimized a mouse model in which hypokalemia combined with myocardial infarction triggered spontaneous ventricular tachycardia in ambulatory mice, and we showed that major leukocyte subsets have opposing effects on cardiac conduction. Neutrophils increased ventricular tachycardia via lipocalin-2 in mice, whereas neutrophilia associated with ventricular tachycardia in patients. In contrast, macrophages protected against arrhythmia. Depleting recruited macrophages in *Ccr2*^*−/−*^ mice or all macrophage subsets with Csf1 receptor inhibition increased both ventricular tachycardia and fibrillation. Higher arrhythmia burden and mortality in *Cd36*^*−/−*^ and *Mertk*^*−/−*^ mice, viewed together with reduced mitochondrial integrity and accelerated cardiomyocyte death in the absence of macrophages, indicated that receptor-mediated phagocytosis protects against lethal electrical storm. Thus, modulation of leukocyte function provides a potential therapeutic pathway for reducing the risk of sudden cardiac death.

Sudden cardiac death occurs when the normal rhythmic depolarization of the myocardium is interrupted, stopping the delivery of oxygenated blood. This condition, which occurs more than 200,000 times in the United States and over 5 million times globally per year, has a survival rate below 10%^1^. The most prevalent underlying pathology is myocardial ischemia, which triggers ventricular tachycardia (VT) or ventricular fibrillation (Vfib). If these arrhythmias are not rapidly treated, then death ensues. Despite this prevalence and lethality, current treatment options are largely limited to defibrillation, which restores normal myocyte depolarization. If a patient survives, secondary prevention relies on improving blood flow and implanting a defibrillator. Although implantable defibrillators can reduce future cardiac mortality, they do not prevent recurrent arrhythmias, and device therapy can impair quality of life. Because it is difficult to predict arrhythmia risk, one-third of patients with a defibrillator never receive an appropriate therapy^2^.

The fundamental electrophysiological mechanisms leading to ventricular arrhythmias have been extensively studied. After myocardial infarction (MI), the normal homogeneous depolarization of the myocardium is replaced by areas of regional heterogeneity that serve as the substrate for re-entry^3^. Various pathologies can propagate re-entry, including abnormal ion channel function^4^, structural changes in ion channels and gap junctions due to oxidation^5^ and genetic disorders. Myocardial fibrosis and dying or dead cells may also slow conduction and, thus, contribute to the arrhythmogenic substrate^6^.

The emerging importance of innate immune cells in the healthy and ischemic heart raises the possibility that leukocytes may contribute to rhythm disorders or help to prevent arrhythmias. Cardiac resident macrophages, with a frequency of 6–8% in the mouse and human heart^7,8^, support normal electrical conduction^9^ and are essential for myocyte energy metabolism^10^. Conditions that increase the risk of an arrhythmia, such as acute MI or myocarditis, are associated with massive changes in myocardial leukocyte numbers and phenotypes^11^.

Given that leukocytes frequently modulate stromal cell and organ functions, we explored, in this study, their contribution to ventricular arrhythmia, a prevalent condition with an unmet therapeutic need. A substantial hurdle for such studies is the dearth of suitable animal models. Although large animals readily develop spontaneous arrhythmia, tools for studying their immune system are limited. By contrast, the mouse offers a plethora of well-developed methods; however, spontaneous VT and Vfib rarely occur. The high heart rate, the small size and distinct action potential have been discussed as reasons for the lack of spontaneous VT^12^. We overcame this challenge with a clinically relevant and surprisingly simple intervention: diet-induced hypokalemia preceding ischemia, which gave rise to recurrent ventricular arrhythmias in conscious, ambulatory mice. With this tool in hand, we then canvassed the contribution of leukocytes to ventricular arrhythmia, starting with neutrophils and macrophages—the most abundant immune cells in the heart.

## Results

### A mouse model of electrical storm.

**Extended Data Fig. 1 |:**
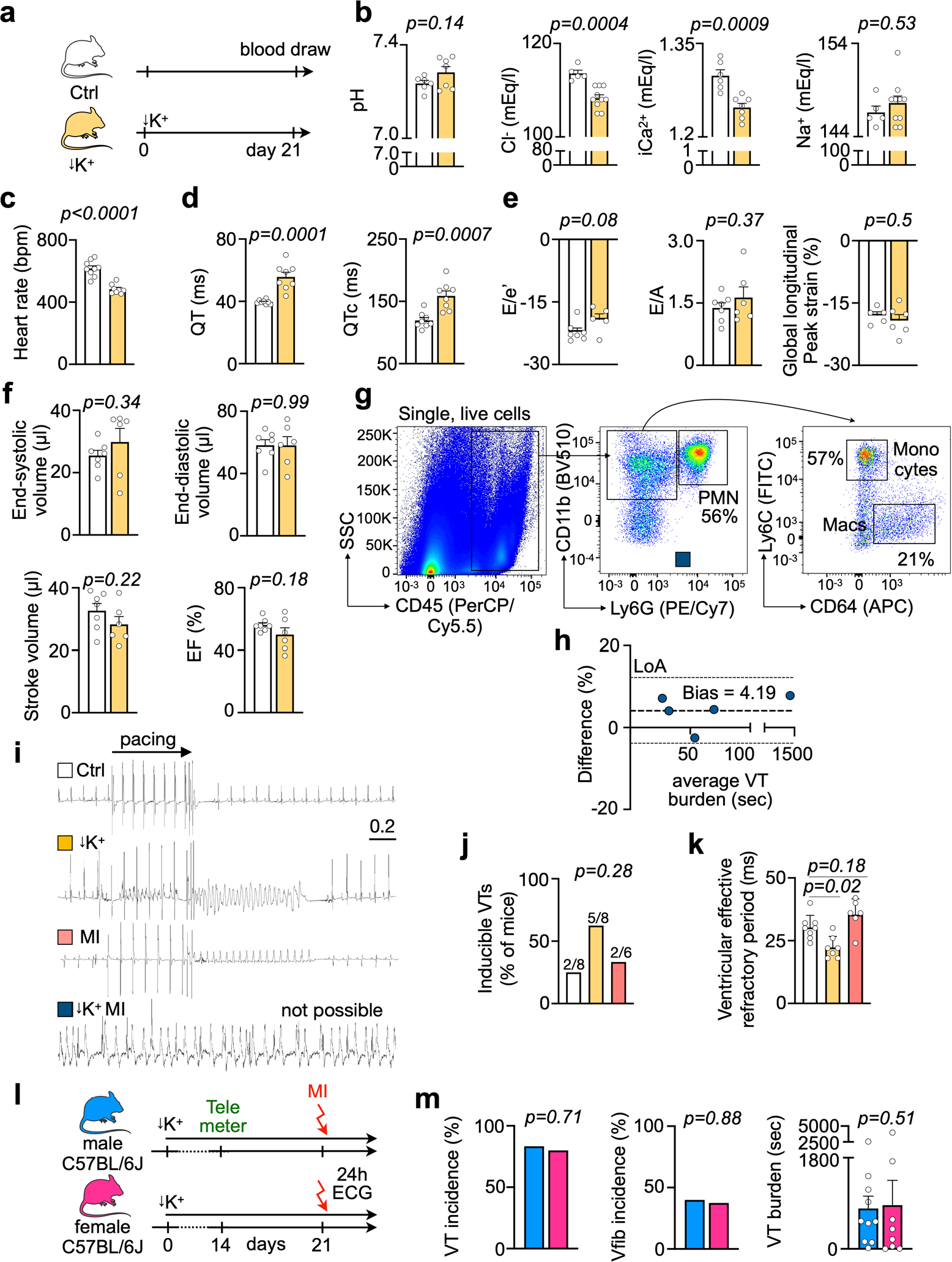
Phenotyping hypokalemic mice. a, Experimental outline. b, pH, Cl^−^, iCa^2+^ and Na^+^ measured in normokalemic (n = 5 or n = 6 mice) and hypokalemic mice (n = 7 or n = 10). Two-sided unpaired t tests were used. c, Average heart rate by telemetric ECG recordings in ambulatory normokalemic (n = 9 mice) and hypokalemic mice (n = 8). Two-sided unpaired t tests were used. d, QT and QTc intervals in normokalemic (n = 8 mice) and hypokalemic mice (n = 8). Two-sided unpaired t tests were used. e, Diastolic function measured by echocardiography in normokalemic (n = 7 mice) and hypokalemic (n = 5 or n = 6) mice indicated by the ratio between mitral inflow velocity and mitral valve annular early diastolic velocity (E/e′), late diastolic trans-mitral flow velocity (E/A) and global longitudinal peak strain (GLS). Two-sided unpaired t tests were used. f, Systolic function by echocardiography in normokalemic (n = 7 mice) and hypokalemic (n = 6) mice, indicated by end-systolic and end-diastolic left ventricular volumes, stroke volume and ejection fraction. Two-sided unpaired t tests were used. g, Gating strategy for cardiac leukocytes. h, Bland-Altman diagram demonstrating the difference of the ventricular tachycardia (VT) burden between two observers in percent. Dashed line: Bias. Dotted lines: Limits of Agreement (LoA). i, Representative ECG tracings from an invasive electrophysiological study. j, Inducibility of ventricular tachycardia (VT) after pacing in naive, hypokalemic and MI mice. Mouse numbers are indicated in plot. MI mice were measured 5 hrs after surgical induction. One-sided Chi-square test was used. k, Ventricular effective refractory period (n = 6 or n = 8 mice per group) in naive, hypokalemic and mice 5 hrs after MI. For STORM mice, catheter insertion caused spontaneous VTs which made an EP study impossible. One-way ANOVA followed by Tukey’s multiple comparisons test were used. l, Experimental outline. m, Incidence of ventricular arrhythmias and VT burden in male (n = 10 mice) and female (n = 8) mice after STORM procedure. Fisher’s exact test (VT and Vfib incidence) and two-sided Mann Whitney test (VT burden) were used. Data are mean ± SEM.

**Fig. 1 |:**
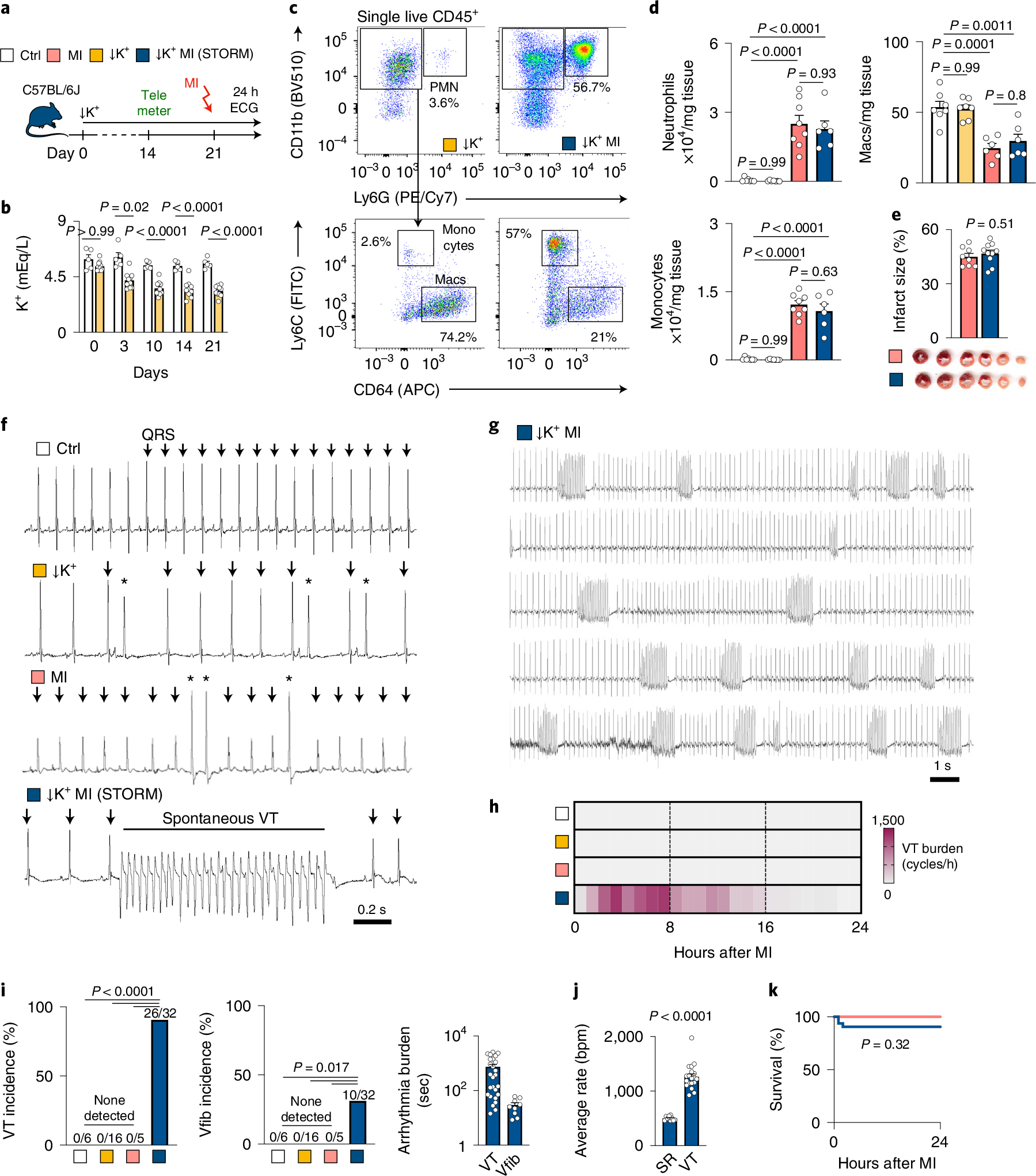
A mouse model of spontaneous electrical storm. **a**, Experimental outline for electrical storm induced by hypokalemia (^↓^K^+^) and MI. ECG recordings are from ambulatory mice within 24 hours after MI. **b**, Plasma potassium levels in control mice (*n* = 5) and mice fed a potassium-deficient diet (*n* = 10). A two-way ANOVA followed by Tukey’s multiple comparisons test was used for statistical analysis. **c**, Flow plots showing neutrophils (PMN), monocytes and cardiac macrophages (Macs) in hearts of hypokalemic and STORM mice. **d**, Quantification of flow cytometry for cardiac neutrophils, monocytes and macrophages in naive mice (*n* = 7), hypokalemic mice (*n* = 7), MI mice (*n* = 8) and STORM mice 24 hours after MI (*n* = 6). A one-way ANOVA followed by Tukey’s multiple comparisons test was used for statistical analysis. **e**, Infarct size in normokalemic (*n* = 8) and STORM (*n* = 10) mice by triphenyltetrazolium chloride (TTC) staining 24 hours after MI. An unpaired two-sided *t*-test was used for statistical analysis. **f**, ECG from telemetric recordings in ambulatory mice. Arrows indicate regular QRS complex; asterisks indicate extrasystoles and VT ventricular tachycardia. **g**, Long ECG strip from a STORM mouse. **h**, Heat map of VT burden expressed as cardiac cycles spent in VT per hour. **i**, VT incidence, Vfib incidence and VT and Vfib burden expressed as seconds spent within 24 hours after MI. Mouse numbers are indicated in brackets. A one-sided chi-square test was used for statistical analysis. **j**, Heart rate during sinus rhythm (SR) and VT expressed as beats per minute (bpm) in STORM mice. A Mann–Whitney test was used for statistical analysis. **k**, Kaplan–Meier survival curve of mice with MI (*n* = 10) and STORM (*n* = 32). A two-sided log-rank Mantel–Cox test was used for statistical analysis. Data are mean ± s.e.m.

Patients with acute MI may develop hypokalemia with serum potassium levels below 3.5 mM due to treatment with diuretics or activation of the sympathetic nervous system^13^. Because the prevalence of life-threatening tachyarrhythmias is inversely correlated to serum potassium levels^13^, we hypothesized that hypokalemic mice develop spontaneous arrhythmias after MI. We tested this hypothesis by feeding C57BL/6J wild-type mice a potassium-deficient diet. Implantation of a telemetric device allowed us to monitor awake mice after infarct induction (Fig. 1a). Providing a potassium-deficient diet for 3 weeks established moderate hypokalemia with accompanying electrolyte disturbances (Fig. 1b and Extended Data Fig. 1a,b). Hypokalemia reduced the resting heart rate and prolonged the QTc time, indicating slower ventricular repolarization (Extended Data Fig. 1c,d). The diastolic and systolic functions as measured by echocardiography were unaltered (Extended Data Fig. 1e,f). Hypokalemia did not affect neutrophil and monocyte recruitment after MI, resident cardiac macrophage disappearance from the ischemic myocardium or infarct size 24 hours after permanent coronary artery ligation (Fig. 1c–e and Extended Data Fig. 1g).

Only occasional ventricular extrasystoles were detected in hypokalemic mice without MI and normokalemic mice with MI, but Spontaneous Tachycardia OccuRred frequently in hypokalemic mice with Myocardial infarction (in the following referred to as STORM; Fig. 1f,g). VT was particularly common during the first day after MI, and most episodes occurred during the first 8 hours (Fig. 1h), a timeline that mirrors the clinical situation^14,15^. VT burden inter-observer variability analysis showed an acceptable bias of 4.2% (Extended Data Fig. 1h). In STORM mice, VT and Vfib reached an incidence of 90% and 31%, respectively (Fig. 1i). The average VT burden of STORM mice was greater than 10,000 cardiac cycles, which translates to over 12 minutes of ventricular arrhythmia (Fig. 1i). Given this prevalence and time course, the VT burden proved a particularly instructive metric in our subsequent studies. Compared to the regular sinus rhythm of STORM mice, the heart rate during VT episodes was twice as fast, well above 1,000 beats per minute (Fig. 1j). STORM mice and normokalemic MI mice had similar survival during the first 24 hours after MI, suggesting that sudden cardiac death is not frequent in wild-type STORM mice (Fig. 1k). Of note, MI carries considerable mortality in mice; however, this mostly takes place several days later^16^.

When we induced VT by rapid pacing in anesthetized mice, arrhythmias were observed in hypokalemic mice without MI and in normokalemic mice with MI. This procedure could not be completed in STORM mice due to frequent recurrent episodes of spontaneous VT, which proved lethal under anesthesia (Extended Data Fig. 1i–k). The arrhythmia burden was similar in male and female STORM mice (Extended Data Fig. 1l,m). In sum, STORM represents a robust model of spontaneous ventricular arrhythmia in awake wild-type mice. We then sought to use the STORM model to investigate the role of key leukocyte populations in arrhythmogenesis.

### Neutrophils incite ventricular arrhythmia.

**Extended Data Fig. 2 |:**
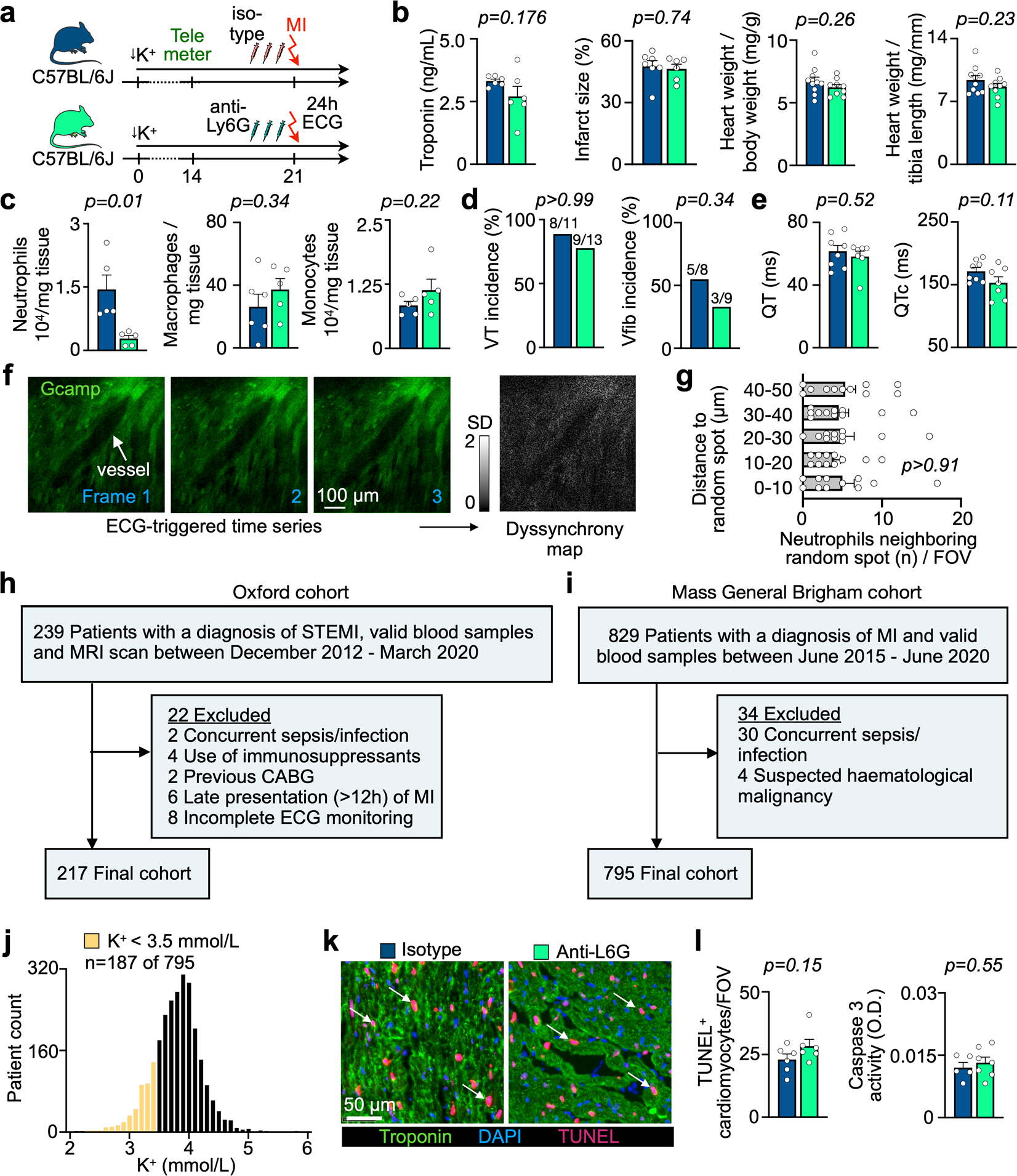
Neutrophil depletion effects after MI. **a**, Experimental outline. **b**, Serum troponin, infarct size, heart weight-to-body weight ratio and heart weight-to-tibia length ratio from isotype-injected (n = 6 or n = 10 mice) and anti-Ly6G antibody-injected (n = 6 or n = 9) mice. Two-sided unpaired t tests were used. **c**, Flow cytometric quantification of cardiac macrophages in STORM mice undergoing isotype (n = 6 mice) or anti-Ly6G antibody (neutrophil depletion, n = 5 mice) injections after MI. Two-sided unpaired t test was used. **d**, Ventricular tachycardia (VT) incidence, ventricular fibrillation (Vfib) incidence (n-numbers indicated in plots) obtained by telemetric ECG recordings in ambulatory isotype-injected (n = 8 mice) and anti-Ly6G-injected (n = 7) mice, all after STORM exposure. Two-sided Fisher’s exact tests was used. **e**, QT and QTc intervals assessed in STORM mice (n = 10 mice) and STORM mice undergoing neutrophil depletion (n = 7). Two-sided unpaired t tests were used. **f**, Confocal microscopy image of isolated Langendorff heart from a control *Myh6-GCaMP8* mouse. The ECG-triggered time series was used to calculate a dyssynchrony map (standard deviation of the Ca^2+^ channel (gcamp) over time). None were identified in this control experiment. Scale bar indicates 100 μm. This experiment was repeated independently four times. **g**, Distances from anti-Ly6G-labeled neutrophils to randomly distributed spots in individual fields of view (FOVs). Data were generated from n = 3 mice and n = 11 FOVs. Three random spots were assigned in each FOV and the distance to neutrophils closer than 50 μm was measured. One-way ANOVA was used. **h**, Exclusion chart for Oxford cohort. **i**, Exclusion chart for Mass General Brigham cohort. **j**, Serum potassium levels measured in MI patients (n = 795) in the Mass General Brigham cohort. Data represent the lowest potassium level measured during hospitalization due to MI. **k**, TUNEL, troponin and DAPI staining of sections from the infarct 5 hrs after MI in STORM mice. Scale bar indicates 50 μm. **l**, Analysis of TUNEL^+^ myocytes in STORM mice injected with isotype (n = 6) or anti-Ly6G neutrophil depleting antibody (n = 6). Caspase 3 activity measured in infarcted myocardium (5 hrs post MI) from isotype control antibody injected C57BL/6 mice (n = 6 mice) and mice with neutrophil depletion treatment (n = 7), all after STORM procedure. Two-sided unpaired t tests were used. Data are mean ± SEM.

**Fig. 2 |:**
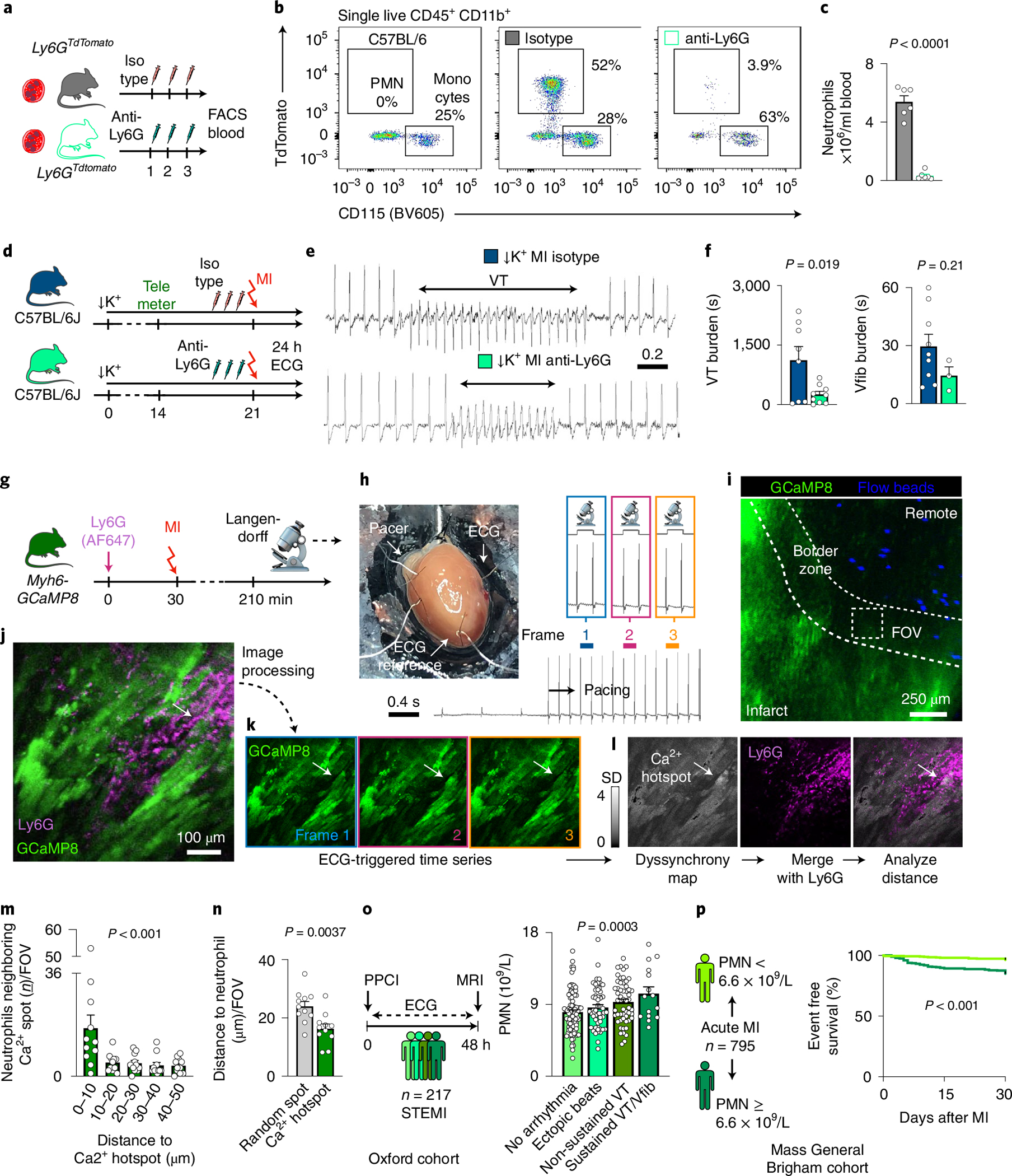
Neutrophils promote ventricular arrhythmia. **a**, Experimental outline for *Ly6G*^*Tdtomato*^ mice with neutrophil depletion, numbers indicate days. **b**, Flow cytometry for blood neutrophils (PMN) and monocytes in *Ly6G*^*Tdtomato*^ mice undergoing isotype or anti-Ly6G antibody injections. **c**, Blood neutrophil counts in isotype (*n* = 6) and anti-Ly6G depleting antibody (*n* = 7) injected mice. A two-sided unpaired *t*-test was used. **d**, Experimental outline for neutrophil depletion in STORM mice, numbers indicate days. **e**, ECG recordings in STORM mice treated with isotype or anti-Ly6G antibody. **f**, VT burden and Vfib burden detected during 24 hours after MI (isotype: *n* = 8, anti-Ly6G: *n* = 9). A two-sided unpaired *t*-test was used. **g**, Experimental design. Langendorff-perfused hearts from *Myh6-GCaMP8* mice were injected with anti-Ly6G (AF647) to label neutrophils, 3 hours after MI. **h**, Original ECG recordings indicating imaging acquisition protocol. **i**, Confocal microscopy of infarct border zone. Blood flow determination beads separate the remote from the infarct zone. Scale bar, 250 μm. **j**, Cardiomyocyte with asynchronous Ca^2+^ signal (arrow) surrounded by Ly6G^+^ neutrophils. Scale bar, 100 μm. This experiment was repeated independently four times. **k**, Time series of Ca^2+^ signal used to identify Ca^2+^ hotspots. The arrow indicates an asynchronous myocyte (see also Supplementary Video 1). **l**, Dyssynchrony map reveals asynchronous cardiomyocytes and was merged with the Ly6G channel. **m**, Distances from neutrophils to Ca^2+^ hotspots (*n* = 11 FOVs from *n* = 3 mice). A one-way ANOVA was used. **n**, Distance of neutrophils to a random spot (gray bar) or a Ca^2+^ hotspot (green). A two-sided unpaired *t*-test was used. **o**, Neutrophil counts from patients with STEMI and PPCI in the Oxford cohort (*n* = 217) were stratified across the following groups: (i) no arrhythmia (*n* = 91 patients), (ii) ventricular ectopic beats (*n* = 57 patients), (iii) non-sustained VT (*n* = 54 patients) and (iv) sustained VT or Vfib (*n* = 15 patients). A Jonkheere–Terpstra test was used. **p**, Patients with acute MI (*n* = 795, Mass General Brigham cohort) were dichotomized at the median neutrophil count and followed for a composite outcome of death or cardiac arrest. A Gehan–Breslow–Wilcoxon test was used. Data are mean ± s.e.m. FACS, fluorescence-activated cell sorting.

We began by investigating neutrophils, which accumulate in the infarct within minutes of ischemia onset and reach their peak abundance 24 hours later^11^. The diverse roles of neutrophils after MI have been described in great detail, but it remains unclear whether they contribute to ventricular arrhythmias. Hence, we depleted circulating neutrophils with antibody injections against neutrophil surface markers (Fig. 2a)^17^. To exclude the effects of neutrophil depletion on infarct size, which influences the occurrence of arrhythmia^18^, we opted for permanent coronary artery occlusion without re-perfusion. Antibody treatment sufficiently depleted neutrophils in STORM mice, whereas monocyte and macrophage populations, blood troponin, 24-hour infarct size and heart weight remained unaffected (Fig. 2a–c and Extended Data Fig. 2a–c). Notably, neutrophil depletion lowered the VT burden when compared to STORM mice with regular neutrophil counts, indicating that neutrophils promote ventricular arrhythmias (Fig. 2d–f and Extended Data Fig. 2d). Neutrophil depletion did not affect the repolarization parameter QTc time (Extended Data Fig. 2e).

To investigate whether neutrophils accumulate in myocardial areas with disturbed conduction, we applied intravital microscopy to beating Langendorff-perfused hearts from *Myh6-GCaMP8* mice (Fig. 2g). *Myh6-GCaMP8* mice express a sensitive Ca^2+^ indicator GCaMP8 under control of the cardiomyocyte-specific *Myh6* promoter, which increases green fluorescent protein (GFP) signal when cytosolic calcium binds to GCaMP8 during systole. ECG-gated imaging of paced isolated hearts focused on the infarct border zone, which we identified with fluorescent beads indicating blood flow (Fig. 2h,i). In every location, we recorded a time-lapse series in which each image frame covered the same portion of the cardiac cycle. We compared these temporal data points to each other by generating a fluorescence intensity standard deviation map (Fig. 2j–l). In *Myh6-GCaMP8* mice without MI, this procedure resulted in homogeneously low standard deviation values across the field of view (FOV), given that all cardiomyocytes cycled synchronously (Extended Data Fig. 2f). In contrast, in mice with MI, we detected regional Ca^2+^ signal inhomogeneity, revealing myocytes in which the timing of peak cytosolic Ca^2+^ concentrations differed from surrounding cells and shifted from cycle to cycle (Fig. 2j–l and Supplementary Videos 1 and 2). Merging standard deviation maps (which we considered ‘dyssynchrony’ maps) with a spectrally resolved acquisition channel reporting on Ly6G^+^ neutrophils, we explored the regional association of neutrophils with dyssynchronous cardiomyocytes. Neutrophils assembled in the vicinity of dyssynchronous cardiomyocytes, whereas randomly placed spots showed no such association (Fig. 2m,n and Extended Data Fig. 2g), implicating neutrophil proximity to heterogeneously depolarizing cardiomyocytes.

To assess the relevance of neutrophils in the clinical setting, we first retrospectively studied patients with ST-elevation myocardial infarction (STEMI) who underwent primary percutaneous coronary intervention (PPCI) and subsequent continuous ECG monitoring for up to 48 hours at Oxford University Hospitals (*n* = 217, Oxford cohort; Fig. 2o, Extended Data Fig. 2h and Extended Data Table 1). We stratified this patient cohort using a prospectively defined arrhythmia score: (i) no arrhythmias, (ii) ventricular ectopic beats, (iii) non-sustained VT and (iv) sustained VT or Vfib. A higher circulating neutrophil count was associated with an increased risk of early VT or Vfib (*P* = 0.0003 for trend across the a priori ordered groups; Fig. 2o). In a logistic regression model (Methods), a higher neutrophil count remained associated with the composite outcome of non-sustained VT, sustained VT or Vfib, even when adjusting for the acute infarct size (measured using magnetic resonance imaging (MRI)) and other factors known to alter the risk of ventricular arrhythmia after MI, including ischemic time and prior beta-blocker use (for each 1 × 10^9^ per L increase in neutrophil count: hazard ratio (HR) = 1.20, 95% confidence interval (CI): 1.06–1.36, *P* = 0.003). In a separate retrospective analysis, we studied the link between circulating neutrophils and clinical outcomes in a cohort of patients with acute MI (both STEMI and NSTEMI, *n* = 795, Mass General Brigham cohort; Extended Data Fig. 2i and Extended Data Table 2), in which ventricular arrhythmia is a leading cause of death^19^. A high neutrophil count, dichotomized at the median of 6.6 × 10^9^ per L for this cohort, was associated with a four-fold elevated risk of cardiac arrest or death at 30 days (HR = 4.5, 95% CI: 2.5–8.1, *P* < 0.001; Fig. 2p). The association with 30-day death or cardiac arrest remained significant in a series of Cox regression analyses controlling for covariates, including age, sex, peak troponin level, monocyte count and creatinine (Extended Data Table 3). Of note, 24% of patients in the Mass General Brigham cohort experienced hypokalemia with a potassium level below 3.5 mmol L^−1^ (Extended Data Fig. 2j), indicating that the parallel observations in the STORM mouse model and in infarct patients may carry clinical relevance. Taken together, these clinical data link blood neutrophil expansion with the occurrence of ventricular arrhythmias and adverse prognosis in patients with MI, matching the causal relationship observed in the ischemic myocardium of STORM mice. However, we acknowledge that our retrospective analyses of both human cohorts are limited by potential confounding from unmeasured risk factors for ventricular arrhythmia. In the Mass General Brigham cohort, it was not possible to ascertain cardiac rhythm at the time of death; hence, non-arrhythmic causes of death are likely to have contributed to the reported outcome.

### Neutrophil-derived lipocalin-2 is pro-arrhythmic.

**Extended Data Fig. 3 |:**
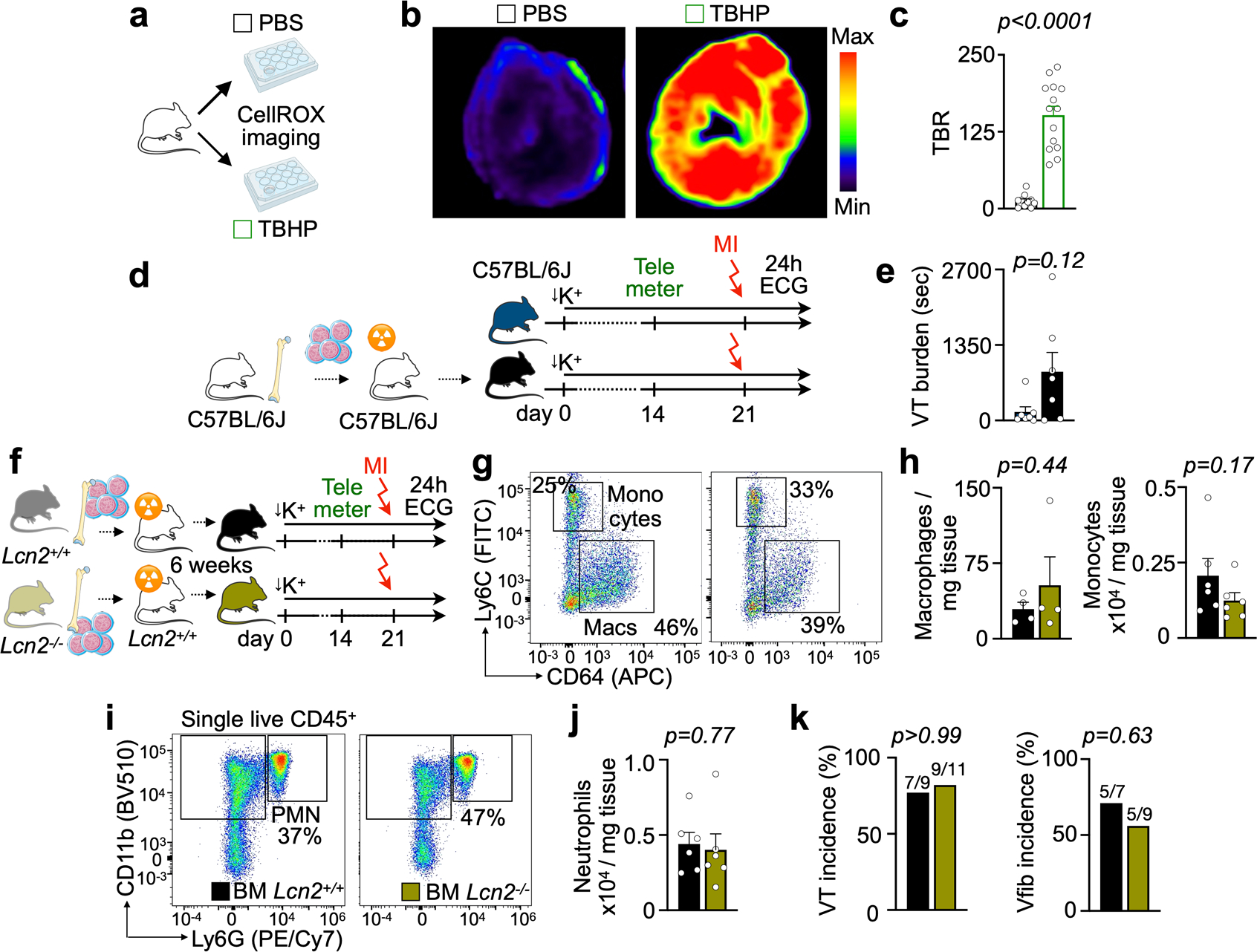
Lcn2 deletion in bone marrow cells. **a**, Experimental outline for CellROX validation. Mice were intravenously injected with CellROX. After 30 min, hearts were excised and cardiac slices were incubated either in PBS or a tert-butyl hydroxyperoxide (TBHP) solution that increases oxidative stress. **b**, Fluorescence images from cardiac short axis slices after intravenous injection of CellROX and incubation with either control PBS or TBHP. **c**, Quantification of target-to-background ratio (TBR) from FRI. Background mean fluorescence intensity was measured in the image background outside tissue. Data are from incubation with PBS (n = 4 mice) or TBHP (n = 4). Each dot represents a cardiac slice. Two-sided unpaired t test was used. **d**, Experimental outline. Lethal irradiation was followed by bone marrow transplantation and the STORM procedure. **e**, Ventricular tachycardia (VT) burden in mice without (n = 7 mice) and with bone marrow transplantation (n = 7), after STORM procedure. Two-sided Mann Whitney test was used. **f**, Experimental outline of bone marrow transplantation approach using wild type or Lcn2^−/−^ donor mice followed by STORM procedure. **g**, Flow plots of cardiac monocyte and macrophage populations in *Lcn2*^*+/+*^ and *Lcn2*^−*/*−^ bone marrow chimeras after STORM procedure, 5hrs after MI. **h**, Quantification of cardiac monocyte and macrophages in *Lcn2*^*+/+*^ (n = 6 mice) and *Lcn2*^*−/−*^ bone marrow chimeras (n = 6) after STORM protocol. Two-sided unpaired t tests were used. **i**, Flow plots of cardiac neutrophils (PMN) in *Lcn2*^*+/+*^ and *Lcn2*^−*/*−^ bone marrow chimeras after STORM procedure, 5hrs after MI. **j**, Quantification of cardiac monocyte and macrophages in *Lcn2*^*+/+*^ (n = 6 mice) and *Lcn2*^−*/*−^ bone marrow chimeras (n = 6) after STORM protocol. A Mann Whitney test (monocytes) and a two-sided unpaired t test (Macrophages) were used. **k**, Ventricular tachycardia (VT) incidence and ventricular fibrillation (Vfib) incidence (n-numbers indicated in plots) in ambulatory *Lcn2*^*+/+*^ (n = 7 mice) and *Lcn2*^−*/*−^ bone marrow chimeras (n = 9) after STORM procedure. Two-sided Fisher’s exact tests were used. Data are mean ± SEM.

**Fig. 3 |:**
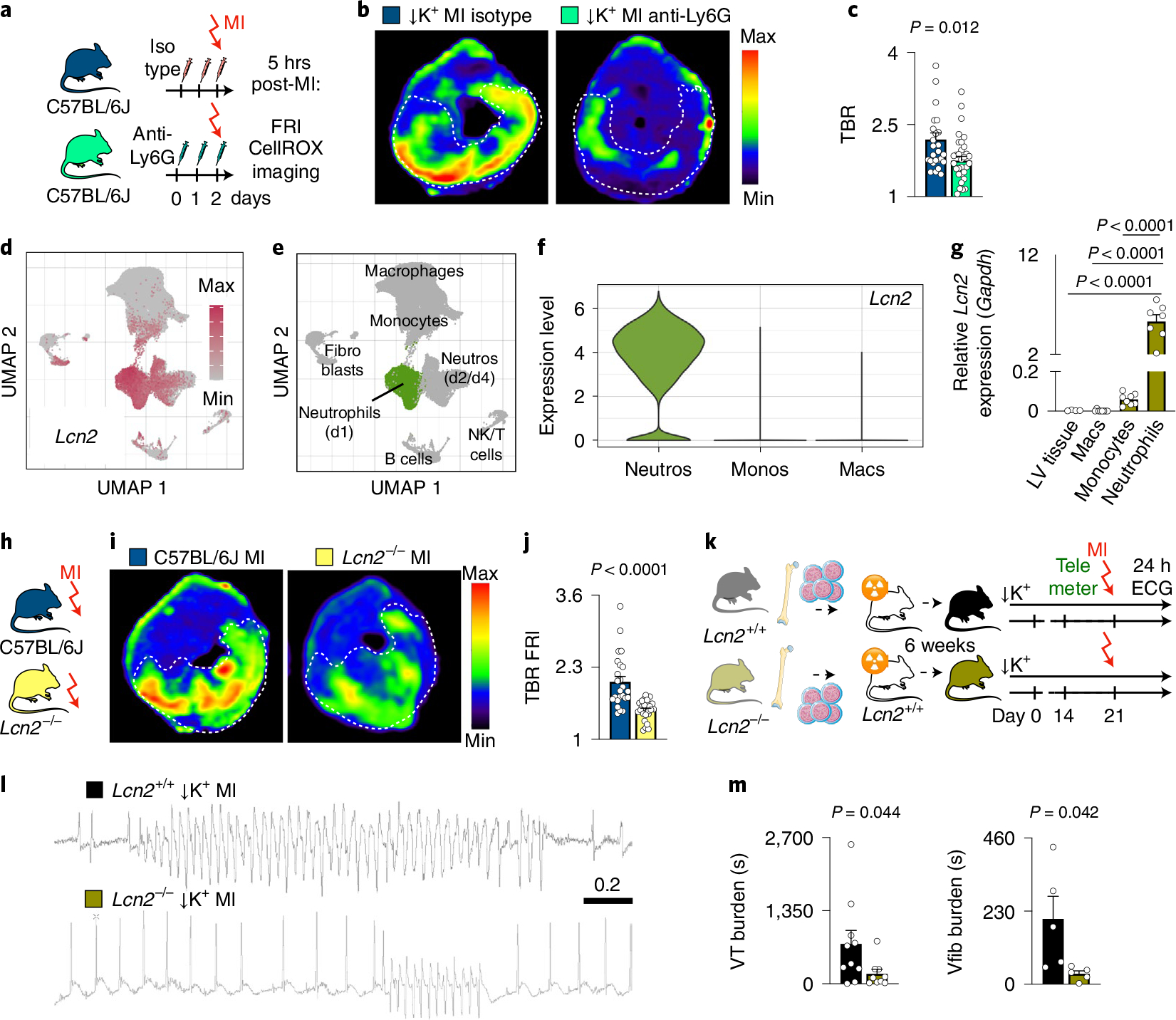
Neutrophil Lcn2 promotes ventricular arrhythmia. **a**, Experimental outline. FRI of ROS in hearts 5 hours after MI using the CellROX imaging agent. **b**, Fluorescence images from cardiac short axis slices after injection of CellROX. **c**, Quantification of TBR from FRI. Data are from isotype antibody-injected controls (*n* = 6 mice) and neutrophil-depleted mice (*n* = 7). Each dot represents a cardiac slice. A two-sided Mann–Whitney test was used for statistical analysis. **d**, scRNA-seq data obtained in three mice after MI. Uniform manifold approximation and projection (UMAP) for dimension reduction indicates *Lcn2* expression and (**e**) cell population identities. **f**, Violin plots indicating *Lcn2* expression in neutrophils, monocytes and macrophages after MI (*n* = 3 mice 24 hours after MI, *n* = 3 mice 48 hours after MI and *n* = 8 mice 4 days after MI; FDR < 0.001). **g**, *Lcn2* expression by quantitative PCR in ischemic myocardium (*n* = 4 mice), flow-isolated macrophages (Macs) (*n* = 8), monocytes (Monos) (*n* = 8) and neutrophils (Neutros) (*n* = 7) 5 hours after MI. *Lcn2* expression was normalized to *Gapdh*. A one-way ANOVA followed by Tukey’s multiple comparisons test was used for statistical analysis. **h**, Experimental outline. FRI of ROS in *Lcn2-*deficient mice and *Lcn2*^*+/+*^ controls 5 hours after MI using CellROX sensor. **i**, Fluorescence images after intravenous CellROX injection. **j**, Quantification of TBR from FRI. Data are from *Lcn2*^*+/+*^ (*n* = 6) and *Lcn2*^*−/−*^ (*n* = 4) mice. Each dot represents a cardiac slice. A two-sided Mann–Whitney test was used for statistical analysis. **k**, Experimental outline. Bone marrow donors were either wild-type (*Lcn2*^*+/+*^) or Lcn2^*−/−*^ mice. Transplant recipients were wild-type mice that subsequently underwent STORM protocol. **l**, ECG recordings from STORM *Lcn2*^*+/+*^ control and *Lcn2*^*−/−*^ bone marrow chimeras. **m**, VT burden and Vfib burden (*Lcn2*^*+/+*^ control, *n* = 8 mice; *Lcn2*^*−/−*^ *n* = 9 mice) within 24 hours after MI. A two-sided Mann–Whitney test (VT burden) and two-sided unpaired *t*-test (Vfib burden) was used for statistical analysis. Data are mean ± s.e.m. FDR, false discovery rate; LV, left ventricular; NK, natural killer.

We next sought to investigate the mechanistic link between neutrophils and VT. We first tested if neutrophil depletion reduces ischemic cell death but found similar numbers of TUNEL^+^ myocytes and caspase-3 activity in the infarcts of STORM mice with reduced and normal neutrophil counts at a time when arrhythmia was most prevalent (Extended Data Fig. 2k,l). We then explored whether neutrophils promote post-MI arrhythmia via reactive oxygen species (ROS)^20^. Five hours after coronary ligation, a fluorescent ROS imaging sensor, which we validated for the specific experiment (Extended Data Fig. 3a–c), was enriched in the infarct, although to a lesser degree if neutrophils were depleted (Fig. 3a–c). We next interrogated available single-cell RNA sequencing (scRNA-seq) data for ROS-generating pathways in neutrophils^21^, comparing expression patterns to those of monocytes and macrophages isolated from mice with acute MI. Interestingly, the third most differentially regulated gene in neutrophils was *Lipocalin-2* (*Lcn2*) (Fig. 3d–f and Supplementary Table 1). Neutrophils rely on Lcn2 to generate ROS while fighting bacteria^22^. However, this defense mechanism can become deleterious, damaging ischemic cardiomyocytes^23,24^. Serum LCN2, also known as neutrophil gelatinase-associated lipocalin (NGAL), increases in patients with MI and heart failure and predicts infarct mortality and adverse outcomes^25^. Motivated by these clinical data and by prior work linking ROS to arrhythmia^20^, we hypothesized that neutrophils may trigger post-MI VT via Lcn2-related mechanisms.

Using quantitative RT–PCR in sorted immune cells, we verified that neutrophils express *Lcn2* at high levels when post-MI arrhythmias peak (Fig. 3g). Imaging in *Lcn2*^*+/+*^ *and Lcn2*^*−/−*^ mice revealed that Lcn2 indeed promotes higher ROS flux rates in the acute infarct (Fig. 3h–j). To study the pro-arrhythmic relevance of neutrophilderived Lcn2, we prepared *Lcn2*^*+/+*^ *and Lcn2*^*−/−*^ bone marrow chimeras in which only bone-marrow-derived cells lack *Lcn2*. These mice then underwent the STORM procedure (Fig. 3k). Interestingly, we noticed that transplanting wild-type bone marrow into wild-type recipients produced a trend toward increased arrhythmias in STORM mice, implying that such cohorts are the appropriate controls (Extended Data Fig. 3d,e). In line with data obtained after neutrophil depletion, *Lcn2*^*−/−*^ bone marrow chimeras had a lower VT and Vfib burden than *Lcn2*^*+/+*^ bone marrow chimeras, even though overall neutrophil counts were unaffected in cardiac tissue (Fig. 3l,m and Extended Data Fig. 3f–k). These data support that neutrophils promote ventricular arrhythmia, at least partially, via Lcn2, which modulates ROS in the acutely ischemic myocardium.

### Macrophages protect against ventricular arrhythmias.

**Extended Data Fig. 4 |:**
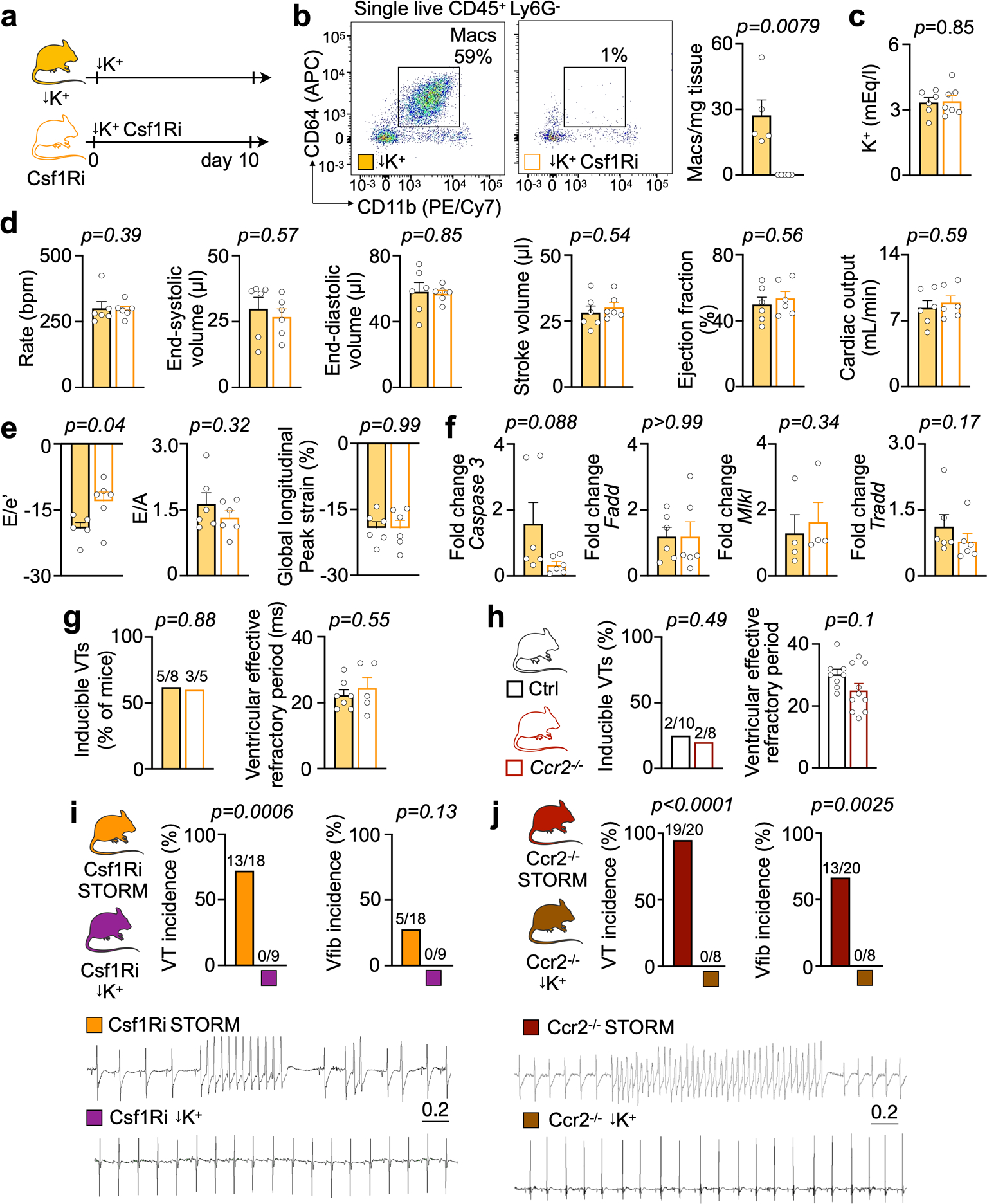
Myeloid cell ablation and steady-state cardiac function. **a**, Experimental outline. **b**, Flow plots and quantification of cardiac macrophages in hypokalemic (n = 5 mice) and hypokalemic mice undergoing macrophage depletion by Csf1Ri (n = 5). Two-sided Mann Whitney test were used. **c**, Serum potassium in hypokalemic (n = 6 mice) and Csf1Ri-treated hypokalemic mice (n = 7). Two-sided unpaired t tests were used. **d**, Systolic function by echocardiography in hypokalemic (n = 6 mice) and Csf1Ri treated hypokalemic mice (n = 6). Two-sided unpaired t tests were used (except for heart rate, two-sided Mann Whitney test). **e**, Diastolic function by echocardiography in hypokalemic (n = 6 mice, except E/e′: n = 5) and Csf1Ri treated hypokalemic mice (n = 6). Ratio between mitral inflow velocity and mitral valve annular early diastolic velocity, E/e′; late diastolic trans-mitral flow velocity, E/A; global longitudinal peak strain, GLS. Two-sided unpaired t tests were used. **f**, *Caspase 3*, *Fadd*, *Mlkl* and *Tradd* expression measured by quantitative PCR in hypokalemic (n = 4 or n = 6 mice, respectively) and Csf1Ri treated hypokalemic mice (n = 4 or n = 6 mice, respectively). Data from macrophage depletion group was normalized to data from hypokalemic mice. Two-sided unpaired t tests were used (except *Mlkl* and *Tradd*, Mann Whitney test). **g**, Ventricular tachycardia (VT) inducibility and ventricular effective refractory period in an invasive electrophysiological study in hypokalemic (n = 7 mice) and Csf1Ri treated hypokalemic mice (n = 5). Fischer’s exact test (VT inducibility) and unpaired t test were used (ventricular effective refractory period). **h**, VT inducibility and ventricular effective refractory period in an invasive electrophysiological study in hypokalemic (n = 8 mice) and *Ccr2*^*−/−*^ treated hypokalemic mice (n = 10). Fischer’s exact test (VT inducibility) and unpaired t test were used (ventricular effective refractory period). **i**, VT incidence and Vfib incidence in C57BL/6 mice treated with Csf1Ri and undergoing STORM (n = 18 mice) or hypokalemia (n = 9). ECG recordings are from ambulatory mice for 24 hrs. Two-sided Fischer’s exact tests were used. **j**, Ventricular tachycardia (VT) incidence and ventricular fibrillation (Vfib) incidence in *Ccr2*^*−/−*^ mice undergoing STORM (n = 20 mice) or hypokalemia (n = 8). ECG recordings are from ambulatory mice for 24 hrs. Two-sided Fischer’s exact tests were used. Data are mean ± SEM.

Given the profound effects that neutrophils have on post-MI arrhythmias, we suspected that macrophages may exert a similar influence. Coinciding with post-MI arrhythmias, cardiac macrophage numbers and phenotypes change markedly, as resident macrophage death and monocyte recruitment^26^ begin shortly after ischemia onset. We tested how monocytes and macrophages influence post-MI arrhythmias with two different depletion strategies. First, we inhibited the colony-stimulating factor 1 receptor (Csf1R). This receptor promotes resident macrophage survival and myeloid cell proliferation^27^. Ten days of Csf1R inhibition efficiently depleted cardiac macrophages even before MI, whereas serum potassium levels, left ventricular function and expression of cell death-associated genes remained unaffected (Extended Data Fig. 4a–f). The second depletion strategy relied on genetic deletion of the chemokine receptor Ccr2. *Ccr2*^−/−^ mice cannot mobilize monocytes from the bone marrow or recruit macrophages to the infarcted heart^28^.

**Extended Data Fig. 5 |:**
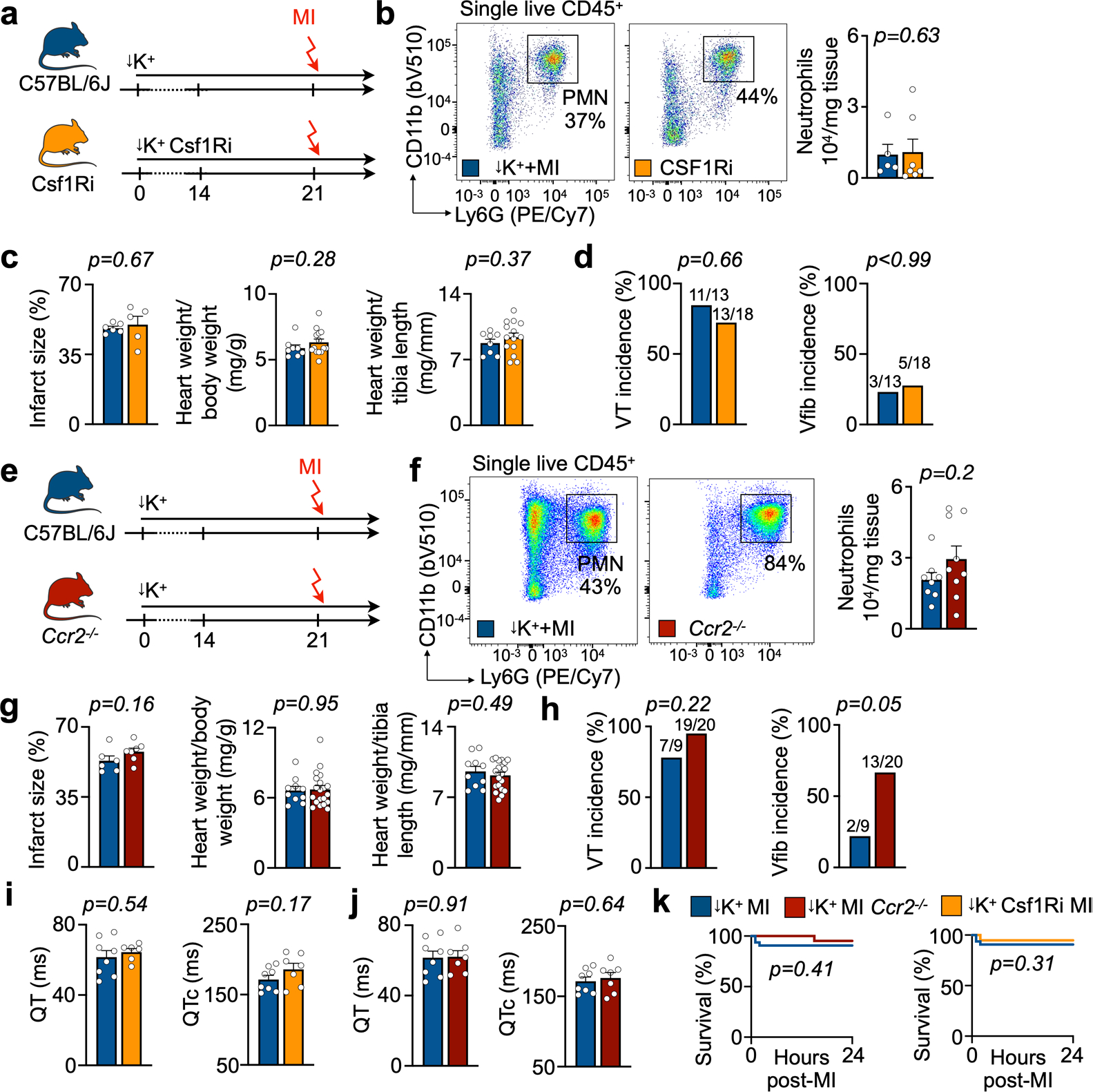
Electrophysiological effects of myeloid cell ablation in. **a**, Experimental outline for macrophage depletion by feeding Csf1Ri PLX5622 via potassium-deficient diet in STORM mice. **b**, Flow plots and quantification of neutrophils (PMN) in STORM mice (n = 5 mice) and mice undergoing Csf1Ri macrophage depletion (n = 7). A two-sided Mann Whitney test was used. **c**, Infarct size, heart-weight-to-body-weight ratio and heart-weight-to-tibia-length ratio in STORM mice (n = 6 or n = 8 mice, respectively) and macrophage-depleted STORM mice (n = 5 or n = 14, respectively). All measurements were done 24hrs post MI. Two-sided unpaired t tests were used. **d**, Ventricular tachycardia (VT) incidence and ventricular fibrillation (Vfib) incidence from telemetric ECG recordings in ambulatory STORM mice (n = 11 mice) and macrophage-depleted STORM mice (n = 12). Two-sided Fischer’s exact tests were used. **e**, Experimental outline for wild type C57BL/6 J and *Ccr2*^*−/−*^ mice undergoing STORM procedure. **f**, Flow plots and quantification of neutrophils (PMN) in C57BL/6 J STORM mice (n = 8) and *Ccr2*^*−/−*^ STORM mice (n = 9). Two-sided unpaired t tests were used. **g**, Infarct size, heart-weight-to-body-weight ratio and heart-weight-to-tibia-length ratio in C57BL/6 J STORM mice (n = 6 or n = 10 mice, respectively) and *Ccr2*^*−/−*^ STORM mice (n = 7 or n = 20, respectively). All measurements were done 24hrs post MI. Two-sided unpaired t tests and Mann Whitney test (heart-weight-to-body-weight ratio) were used. **h**, VT incidence and Vfib incidence obtained by telemetric ECG-recordings in ambulatory C57BL/6 J STORM mice (n = 11 mice) and *Ccr2*^*−/−*^ STORM mice (n = 12). Two-sided Fischer’s exact tests were used. **i**, QT and QTc intervals in STORM mice (n = 10 mice) and STORM mice with Csf1Ri (n = 6–7). Two-sided unpaired t test was used. **j**, QT and QTc intervals in C57BL/6 J STORM mice (n = 10 mice) and *Ccr2*^*−/−*^ STORM mice (n = 7). Two-sided unpaired t test was used. **k**, Kaplan-Meier survival curves of C57BL/6 STORM mice (n = 32 mice), *Ccr2*^*−/−*^ STORM mice (n = 21) and Csf1Ri STORM mice (n = 19). A log-rank Mantel-Cox test was used. Data are mean ± SEM.

**Fig. 4 |:**
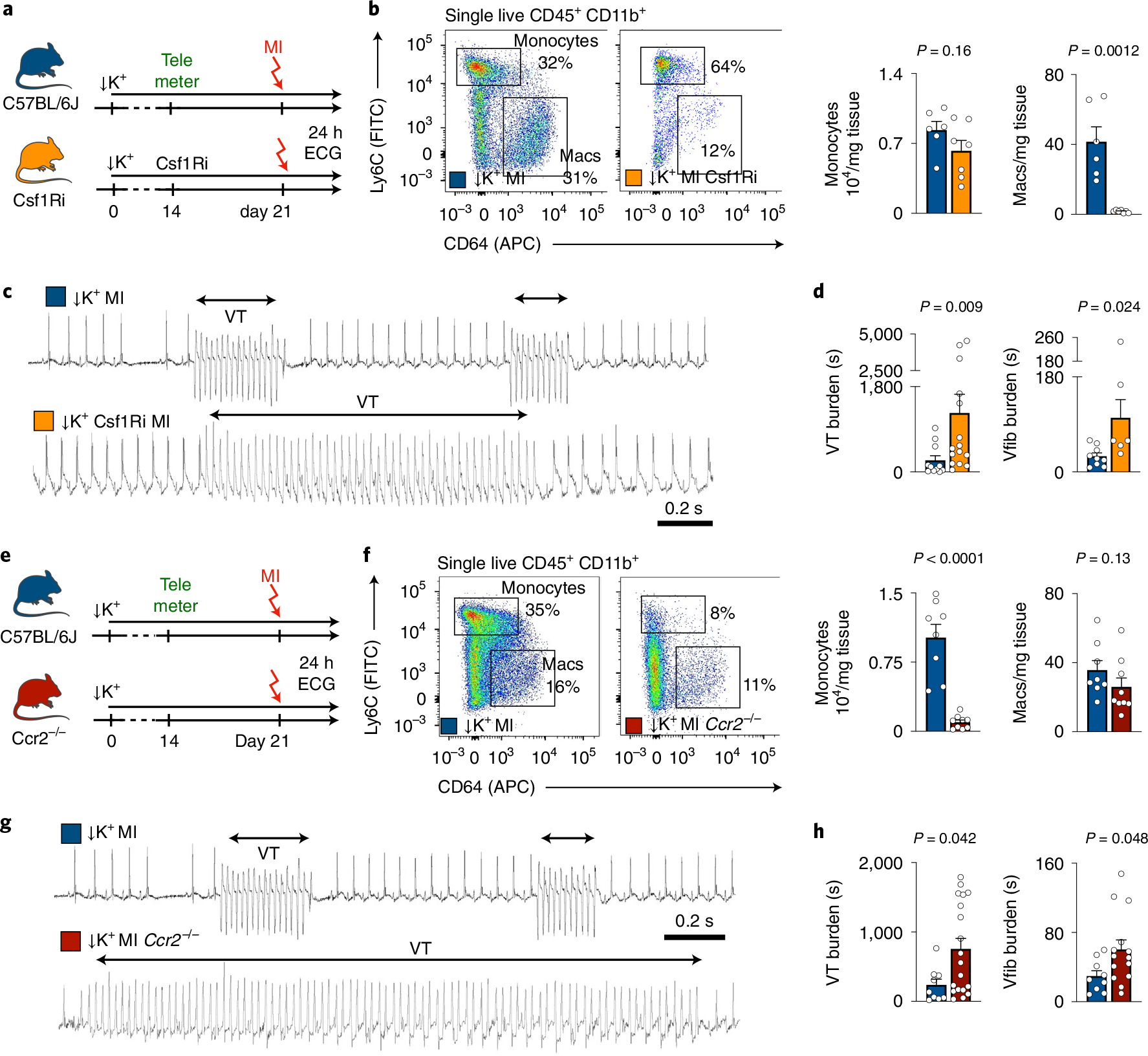
Macrophages protect against ventricular arrhythmia. **a**, Experimental outline for macrophage depletion with Csf1R inhibitor in STORM mice. **b**, Flow plots and quantification of cardiac monocytes and macrophages (Macs) in STORM control mice (*n* = 6) and after macrophage depletion by Csf1Ri (*n* = 7). A two-sided unpaired *t*-test (monocytes) and a two-sided Mann–Whitney test (macrophages) were used for statistical analysis. **c**, ECG recordings from STORM mice with and without macrophage depletion after MI. **d**, VT burden (STORM control mice, *n* = 11; STORM Csf1Ri, *n* = 15) and Vfib burden (STORM control mice, *n* = 6; STORM Csf1R, *n* = 5) within 24 hours after MI. Two-sided Mann–Whitney tests were used for statistical analysis. **e**, Experimental outline. **f**, Flow cytometry for cardiac monocytes and macrophages (Macs) in wild-type STORM control mice (*n* = 8) and *Ccr2*^*−/−*^ STORM mice (*n* = 9). A two-sided unpaired *t*-test (monocytes) and a two-sided Mann–Whitney test (macrophages) were used for statistical analysis. **g**, ECG recordings from wild-type STORM control and *Ccr2*^*−/−*^ STORM mice. **h**, VT burden (wild-type STORM control, *n* = 10 mice; *Ccr2*^*−/−*^ STORM, *n* = 19) and Vfib burden (wild-type STORM control, *n* = 8; *Ccr2*^*−/−*^ STORM, *n* = 14) after MI. Two-sided Mann–Whitney tests were used for statistical analysis. Data are mean ± s.e.m.

In mice without MI, rapid pacing induced similar VT in controls, in mice treated with Csf1R inhibitor (Extended Data Fig. 4g) or in *Ccr2*^*−/−*^ mice (Extended Data Fig. 4h). In hypokalemic mice without MI, macrophage depletion did not induce spontaneous VT or Vfib (Extended Data Fig. 4i,j). We next combined macrophage depletion with the STORM procedure. Csf1R inhibition depleted macrophages after MI (Fig. 4a,b), whereas neutrophil and monocyte counts, infarct size at 24 hours after coronary artery ligation and heart weight remained unaffected (Extended Data Fig. 5a–c). To our surprise, telemetric recordings in STORM mice treated with the Csf1R inhibitor revealed higher VT and Vfib burden compared to STORM controls (Fig. 4c,d and Extended Data Fig. 5d). We then deployed our second strategy—that is, inhibiting macrophage recruitment—in *Ccr2*^*−/−*^ STORM mice. This resulted in fewer infarct monocytes (Fig. 4e,f) presumably because, at early timepoints, these cells have not yet differentiated into macrophages. Neutrophils, infarct size and heart weight were similar to those in wild-type STORM controls (Extended Data Fig. 5e–g). *Ccr2*^*−/−*^ STORM mice had an increased post-MI VT and Vfib burden (Fig. 4g,h and Extended Data Fig. 5h). Macrophage depletion did not affect repolarization, as the QTc time remained unchanged (Extended Data Fig. 5i,j). In STORM mice, macrophage depletion did not alter survival in the first 24 hours after MI (Extended Data Fig. 5k). Altogether, these data suggest that macrophages play a protective role in MI-induced ventricular arrhythmias, irrespective of the cell subset. This insight motivated us to explore how macrophages exert such function in acute MI.

### Macrophage depletion impairs efferocytosis in STORM mice.

**Fig. 5 |:**
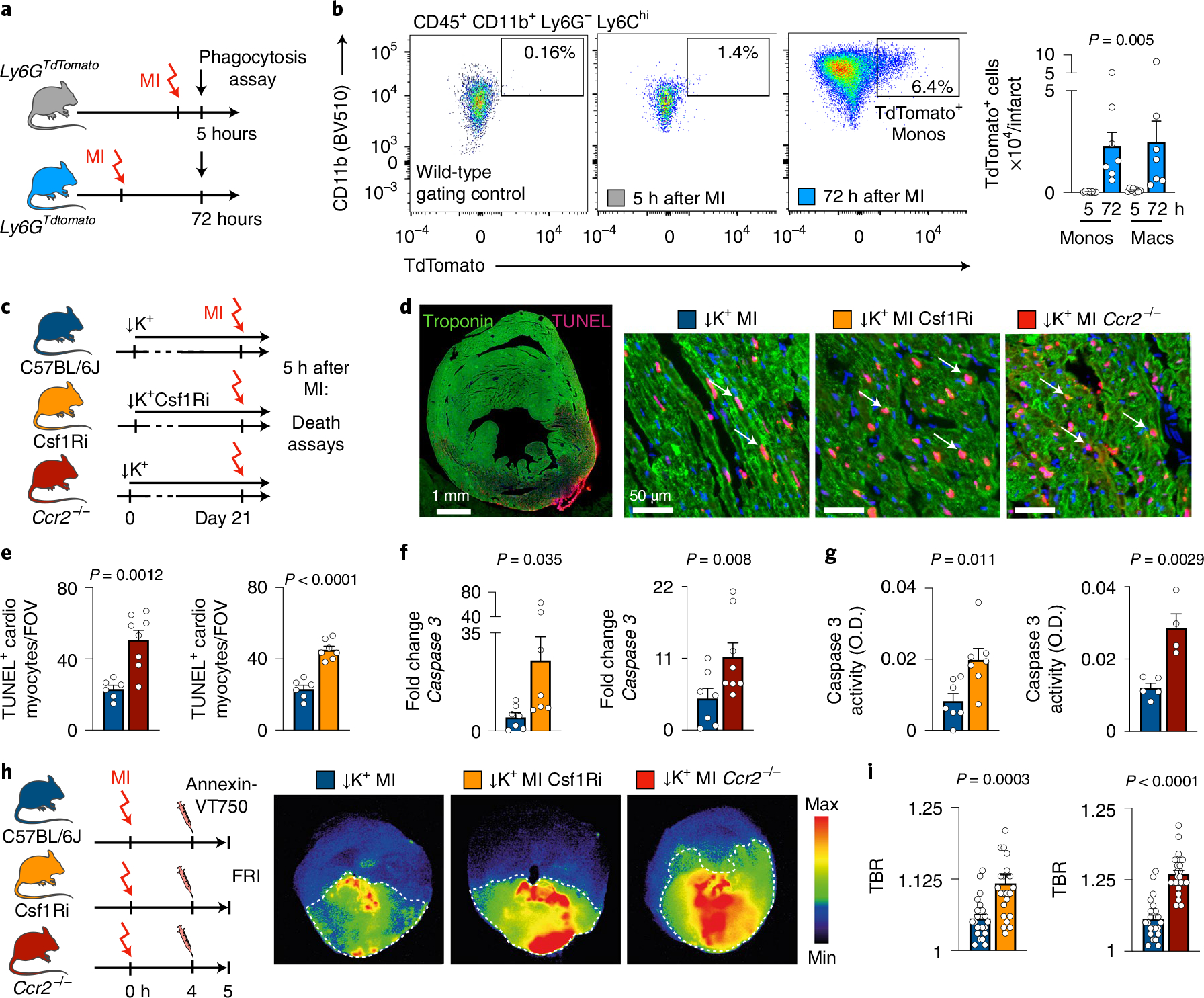
Macrophage depletion accelerates myocyte death and impairs efferocytosis during acute MI. **a**, Experimental outline. **b**, Flow plots after gating for cardiac macrophages (Macs). Bar graphs show quantification of TdTomato^+^ cardiac monocytes (Monos) and TdTomato^+^Macs at 5 hours (*n* = 7 mice) or 72 hours (*n* = 7 mice) after MI. One-way ANOVA followed by Tukey’s multiple comparison’s test was used for statistical analysis. **c**, Experimental outline for wild-type C57BL/6 mice, mice after Csf1Ri macrophage depletion and *Ccr2*^*−/−*^ mice, all after STORM protocol. **d**, TUNEL, troponin and DAPI staining of sections from the infarct region 5 hours after MI. Scale bar, 50 μm. This experiment was repeated independently twice. **e**, Analysis of TUNEL^+^ myocytes in hearts of C57BL/6 mice (*n* = 6), mice treated with Csf1Ri (*n* = 7) and *Ccr2*^*−/−*^ mice (*n* = 8), all after STORM protocol. FOVs were analyzed in the infarct core. Two-sided unpaired *t*-tests were used for statistical analysis. **f**, *Caspase-3* expression by quantitative PCR in infarct tissue from C57BL/6 mice (*n* = 7), mice treated with Csf1Ri (*n* = 7) and *Ccr2*^*−/−*^ mice (*n* = 8), all after STORM protocol. Data from macrophage depletion groups were normalized to data from STORM mice. Two-sided Mann–Whitney tests were used for statistical analysis. **g**, Enzymatic activity of Caspase-3 measured in left ventricular infarct tissue from C57BL/6 mice (*n* = 5 (left) or *n* = 7 (right)), mice treated with Csf1Ri (*n* = 7) and *Ccr2*^*−/−*^ mice (*n* = 5). Two-sided unpaired *t*-tests were used for statistical analysis. **h**, Experimental outline and images from FRI after intravenous injection of Annexin-VT750. **i**, TBR from FRI. Data are from STORM control mice (*n* = 22 slices), mice treated with Csf1Ri (*n* = 23) and *Ccr2*^*−/−*^ mice (*n* = 25). Data are from *n* = 4–5 mice per group. Each dot represents a cardiac slice from an infarcted mouse. Two-sided Mann–Whitney tests were used for statistical analysis. Data are mean ± s.e.m.

Given the opposite effects that we observed for neutrophils and macrophages, and that macrophages are thought to remove short-lived neutrophils from healing infarcts via phagocytosis^29^, we first examined such interaction at the time when post-MI arrhythmia occurs. We employed flow cytometry in neutrophil reporter Ly6G^TdTomato^ mice^30^ to test if macrophages phagocytose neutrophils 5 hours after coronary ligation. As expected, we clearly detected macrophages that had taken up neutrophils on day 3 after MI. However, macrophage-associated TdTomato fluorescence was extremely limited 5 hours after MI (Fig. 5a,b), when ventricular arrhythmias occurred. These data indicated that the anti-arrhythmic functions of macrophages cannot be explained by removal of pro-arrhythmic neutrophils.

Macrophages clear debris and apoptotic cells, a process termed efferocytosis, which is instrumental for tissue repair and infarct healing^31,32^. Depleting macrophages may lead to accumulation of cellular debris and dead cells, which may give rise to heterogeneous conduction velocities or local conduction block, both considered key to re-entry and VT genesis. We, therefore, quantitated TUNEL^+^ cardiomyocytes in the infarcts of STORM controls, STORM mice with Csf1R inhibition and *Ccr2*^*−/−*^ STORM mice at the time of peak VT burden, 5 hours after permanent coronary ligation. As we had hypothesized, TUNEL^+^ cardiomyocytes accumulated more readily in the infarcts of mice in which macrophages had been depleted (Fig. 5c–e).

Accelerated cell death may also cause higher accumulation of dead cells. Even though the final infarct size and survival measured 24 hours after ischemia was similar in STORM control and STORM macrophage depletion cohorts (Extended Data Fig. 5c,g,k), cardiomyocytes may die faster and at earlier timepoints when macrophages are absent, potentially contributing to the peak of arrhythmia prevalence at that time. Indeed, expression levels and activity of caspase-3, a mediator of programmed cell death, were higher in the infarct tissue of Csf1R inhibitor-treated and *Ccr2*^*−/−*^ STORM mice than in STORM controls (Fig. 5f,g). To evaluate cell death with an orthogonal method, we intravenously injected a near-infrared fluorescent Annexin-V molecular imaging probe, which binds to phosphatidyl serine on the surface of apoptotic cells and in the interior of necrotic cells^33^, 4 hours after MI and harvested the hearts 60 minutes later. In all three cohorts, fluorescent reflectance imaging (FRI) of cardiac short axis slices showed binding of the imaging probe primarily in the infarcts. We also found higher target-to-background ratios (TBRs) in the infarcts of Csf1R inhibitor-treated and *Ccr2*^*−/−*^ STORM mice when compared to STORM controls (Fig. 5h,i). From these data, we concluded that, in the absence of macrophages, cardiomyocytes may die at higher rates 5 hours after onset of ischemia. Disrupted removal of dead cells may also contribute to their accumulation.

### Macrophage depletion compromises mitochondrial function after MI.

The accelerated cardiomyocyte demise in mice without macrophages raised the question of how macrophages defend myocytes against ischemic cell death. The primary cause of hypoxic cell death is energy deficit due to disrupted mitochondrial function, and these organelles also induce programmed cell death when under duress^34^. Interestingly, stressed cells may shed mitochondria in relatively large extracellular vesicles^10^. Cardiac macrophages may augment mitochondrial function in cardiomyocytes by scavenging dysfunctional organelles expelled in vesicles called exophers, supporting steady-state cardiomyocyte metabolism and overall organ function^10^. Furthermore, dysfunctional mitochondria give rise to arrhythmia via ROS and ATP deprivation as ~30% of cardiomyocytes’ energy consumption supports ion handling needed for proper excitation^4^.

**Extended Data Fig. 6 |:**
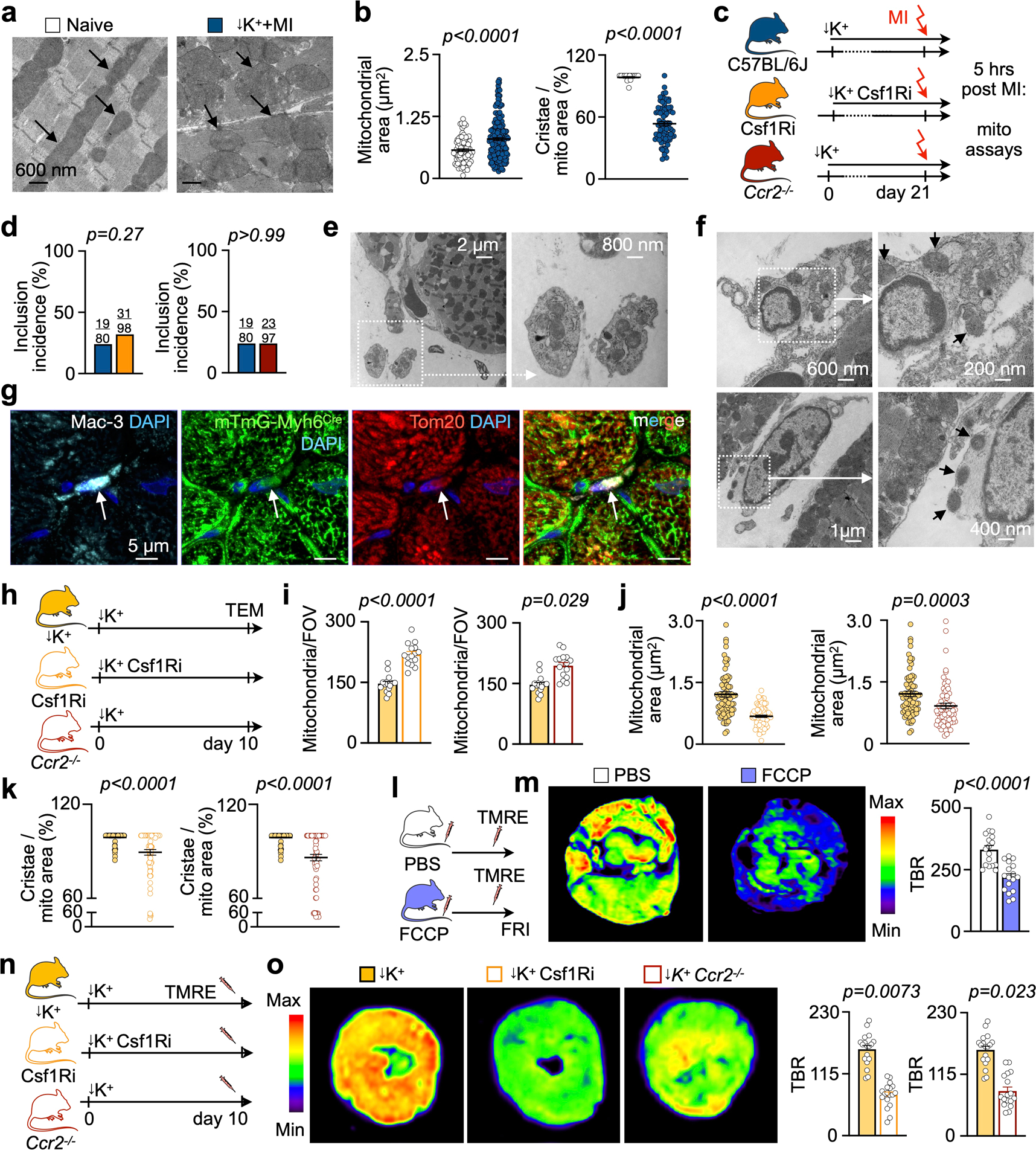
Mitochondrial health after myeloid cell ablation. **a**, Electron microscopy. Arrows indicate mitochondria. Scale bar indicates 600 nm. Experiment was repeated independently three times. **b**, Mitochondrial area and cristae formation in naive C57BL/6 (n = 83 mitochondria (left) or n = 16 (right), n = 1 mouse) and mice undergoing STORM model (n = 178 (left) or n = 80 (right), n = 3 mice, 5 FOVs/mouse). Dots are mitochondria. Two-sided Mann Whitney test (area) and two-sided unpaired t test (cristae) were used. **c**, Experimental outline. **d**, Incidence of paracrystalline inclusions in C57BL/6, Csf1Ri and *Ccr2*^*−/−*^ STORM mice (mouse numbers are indicated in plots). Two-sided unpaired t tests were used. **e**, Exopher containing mitochondria in the extracellular space in the infarct of STORM mice. Scale bar indicates 2 μm or 800 nm. Experiment was repeated independently three times. **f**, Phagocyte with mitochondrial content in the infarct of STORM mouse. Scale bar indicates 600 nm or 200 nm and 1 μm or 400 nm. Experiment was repeated independently three times. **g**, Histological DAPI, mTmG/myh6-GFP, Mac3 and Tom20 staining from infarcts. Circles indicate area of interest. Scale bar indicates 5 μm. Experiment was repeated independently three times. **h**, Experimental outline. **i**, Mitochondrial count per field of view (FOV) in C57BL/6 (n = 15 FOV), mice with Csf1Ri (n = 15) and *Ccr2*^*−/−*^ mice (n = 15). n = 3 mice/group, 5 FOVs/mouse. Nested t tests were used. **j**, Mitochondrial area in wild type mice (n = 80 mitochondria), mice after Csf1Ri (n = 72) and *Ccr2*^*−/−*^ mice (n = 66). n = 3 mice/group; 5 FOVs/mouse. Dots are mitochondria. Two-sided Mann Whitney tests were used. **k**, Percent of cristae area per mitochondrial area in hearts of wild type mice (n = 80 mitochondria), mice after Csf1Ri (n = 72) and *Ccr2*^*−/−*^ mice (n = 66). n = 3 mice/group; 5 FOVs/mouse. Dots are mitochondria. Two-sided Mann Whitney tests were used. **l**, Experimental outline. **m**, Fluorescence images from cardiac short axis slices after injection of PBS or FCCP. Target-to-background ratio (TBR) from FRI. Background mean fluorescence intensity: image background outside tissue. PBS (n = 3 mice) and FCCP (n = 4 mice). Dots are cardiac slices. Unpaired t test was used. **n**, Experimental outline **o**, TBR from FRI. Background fluorescence intensity was measured in image backgrounds. Control mice (n = 18 images), mice treated with Csf1Ri (n = 18 images) and *Ccr2*^*−/−*^ mice (n = 17 images), all without MI. n = 3 mice/group. Dots are cardiac slices. Two-sided unpaired t test was used. Data are mean ± SEM.

**Fig. 6 |:**
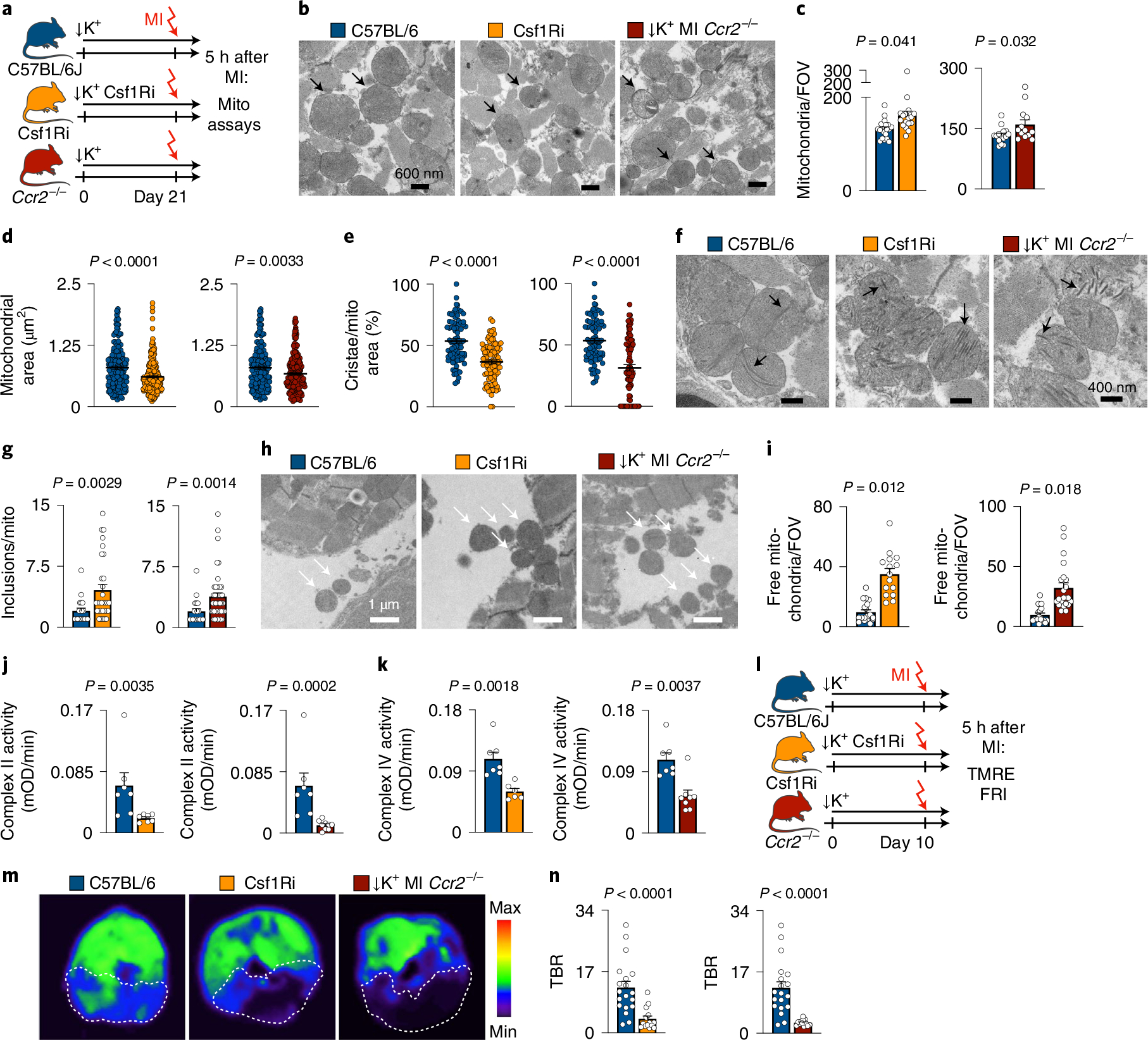
Macrophages preserve mitochondrial integrity after MI. **a**, Experimental outline. **b**, Electron microscopy in infarcts. Arrows indicate mitochondria; scale bar, 600 nm. This experiment was repeated independently three times. **c**, Mitochondrial count per FOV in wild-type mice (*n* = 15 FOVs), mice with Csf1Ri (*n* = 21) and *Ccr2*^*−/−*^ mice (*n* = 13) (*n* = 3 mice per group, 3–5 FOVs per mouse). A two-sided nested *t*-test was used. **d**, Mitochondrial area in infarcts of wild-type mice (*n* = 178 mitochondria), mice after Csf1Ri (*n* = 239) and *Ccr2*^*−/−*^ mice (*n* = 229) (*n* = 3 mice per group; 5 FOVs per mouse). Dots indicate individual mitochondria. A two-sided Mann–Whitney test was used. **e**, Percent cristae area per mitochondrial area in infarcts of wild-type (*n* = 80 mitochondria), mice with Csf1Ri (*n* = 98) and *Ccr2*^*−/−*^ mice (*n* = 87) (*n* = 3 mice per group, 10 FOVs per mouse). Each dot represents a mitochondrion. A two-sided unpaired *t*-test (Csf1Ri) and Mann–Whitney test (*Ccr2*^−/−^) was used. **f**, Electron micrographs of mitochondrial ultrastructure with paracrystalline inclusions (arrows). **g**, Paracrystalline inclusions per mitochondrion in wild-type (*n* = 19 mitochondria), after Csf1Ri macrophage depletion (*n* = 31) and *Ccr2*^*−/−*^ mice (*n* = 44). Dots are mitochondria. A two-sided Mann–Whitney test was used. **h**, Electron micrographs of free mitochondria (arrows) in the extracellular space. Scale bar, 1 μm. This experiment was repeated independently three times. **i**, Free mitochondria in wild-type (*n* = 15 FOVs), mice with Csf1Ri (*n* = 15) and *Ccr2*^*−/−*^ mice (*n* = 19) (*n* = 3 mice per group, 5–7 FOVs per mouse). A two-sided nested *t*-test was used. **j**, Enzymatic activity of mitochondrial complex II in infarcts of wild-type (*n* = 7 mice), after Csf1Ri (*n* = 7) and *Ccr2*^*−/−*^ mice (*n* = 9). A two-sided Mann–Whitney test was used. **k**, Enzymatic activity of mitochondrial complex IV in infarcts of wild-type (*n* = 7 mice), after Csf1Ri (*n* = 6) and *Ccr2*^*−/−*^ (*n* = 8), all after STORM protocol. A two-sided Mann–Whitney test was used. **l**, Experimental outline of TMRE perchlorate imaging. **m**, Representative images of TMRE imaging. **n**, TBR from FRI. STORM mice (*n* = 19 slices), STORM mice with Csf1Ri (*n* = 14) and STORM *Ccr2*^*−/−*^ mice (*n* = 12). Data are from *n* = 3 or *n* = 4 mice per group. Dots indicate cardiac slices. A two-sided Mann–Whitney test was used. Data are mean ± s.e.m.

To explore how macrophage depletion affects mitochondrial health in acute MI, we first performed transmission electron microscopy (TEM) in infarct tissue obtained from STORM mice with and without macrophage depletion, 5 hours after coronary ligation. MI led to mitochondrial swelling and ultrastructure loss, as indicated by reduced cristae (Extended Data Fig. 6a,b). When macrophages were depleted, the number of dysmorphic mitochondria increased in cardiomyocytes (Fig. 6a–c). In mice with macrophage depletion, cardiomyocytes’ mitochondria were smaller, and the area covered by cristae was smaller than in STORM controls, indicating accelerated structural and functional mitochondrial collapse^35^, presumably after rupture (Fig. 6d,e). Paracrystalline inclusions, a hallmark of cellular energy deprivation^36^, were more frequent in mitochondria of macrophage depletion groups (Fig. 6f,g and Extended Data Fig. 6c,d). We next focused on extracellular mitochondria. We observed such mitochondria in two different forms, either freely positioned in the extracellular space (Fig. 6h) or inside vesicles (Extended Data Fig. 6e) that resembled previously described exopher-like structures^10^. In addition, mitochondria were also located adjacent to and inside phagocytic cells (Extended Data Fig. 6f,g). These data suggest that macrophages clear cardiomyocyte-derived mitochondria in STORM mice at 5 hours after MI. In Csf1R inhibitor-treated and *Ccr2*^−/−^ STORM mice, free extracellular mitochondria were more numerous (Fig. 6i), indicating that mitochondrial removal was impaired when macrophages were absent. Of note, some mitochondrial deterioration after macrophage depletion was observed in mice without MI (Extended Data Fig. 6h–k), indicating that mitochondrial compromise preceded ischemia.

On a functional level, macrophage depletion lowered activity of the mitochondrial respiratory chain enzymes succinate dehydrogenase (complex II) and cytochrome c oxidase (complex IV) (Fig. 6j,k). Enzymes of the mitochondrial respiratory chain pump protons across the inner mitochondrial membrane, producing a transmembrane electrical potential gradient (**ΔΨm**) that is essential for ATP synthesis^37^. In conditions of cell death and compromised mitochondrial integrity, the **ΔΨm** diminishes^38^. To test for loss of mitochondrial **ΔΨm** in macrophage depletion cohorts, we intravenously injected tetramethylrhodamine ethyl ester (TMRE), a positively charged lipophilic fluorescent imaging agent that accumulates inside negatively charged mitochondria. Fifteen minutes after TMRE injection, we imaged myocardial short axis slices ex vivo. In a pilot imaging experiment, treatment with a mitochondrial uncoupler, which reduces **ΔΨ**^39^, changed the myocardial TMRE accumulation accordingly, thereby validating this imaging assay (Extended Data Fig. 6l,m). Macrophage depletion with Csf1R inhibition and in *Ccr2*^*−/−*^ mice resulted in decreased myocardial fluorescence signal in non-infarcted hearts (Extended Data Fig. 6n,o) and also in the infarcted myocardium (Fig. 6l–n), suggesting that macrophages may preserve cardiomyocytes’ mitochondrial **ΔΨm**. These imaging data accord with reduced activity of mitochondrial complex II and IV observed after macrophage depletion, collectively supporting the concept of impaired mitochondrial integrity and function in macrophage-depleted myocardium.

### Phagocytosis receptor function prevents sudden cardiac death.

**Extended Data Fig. 7 |:**
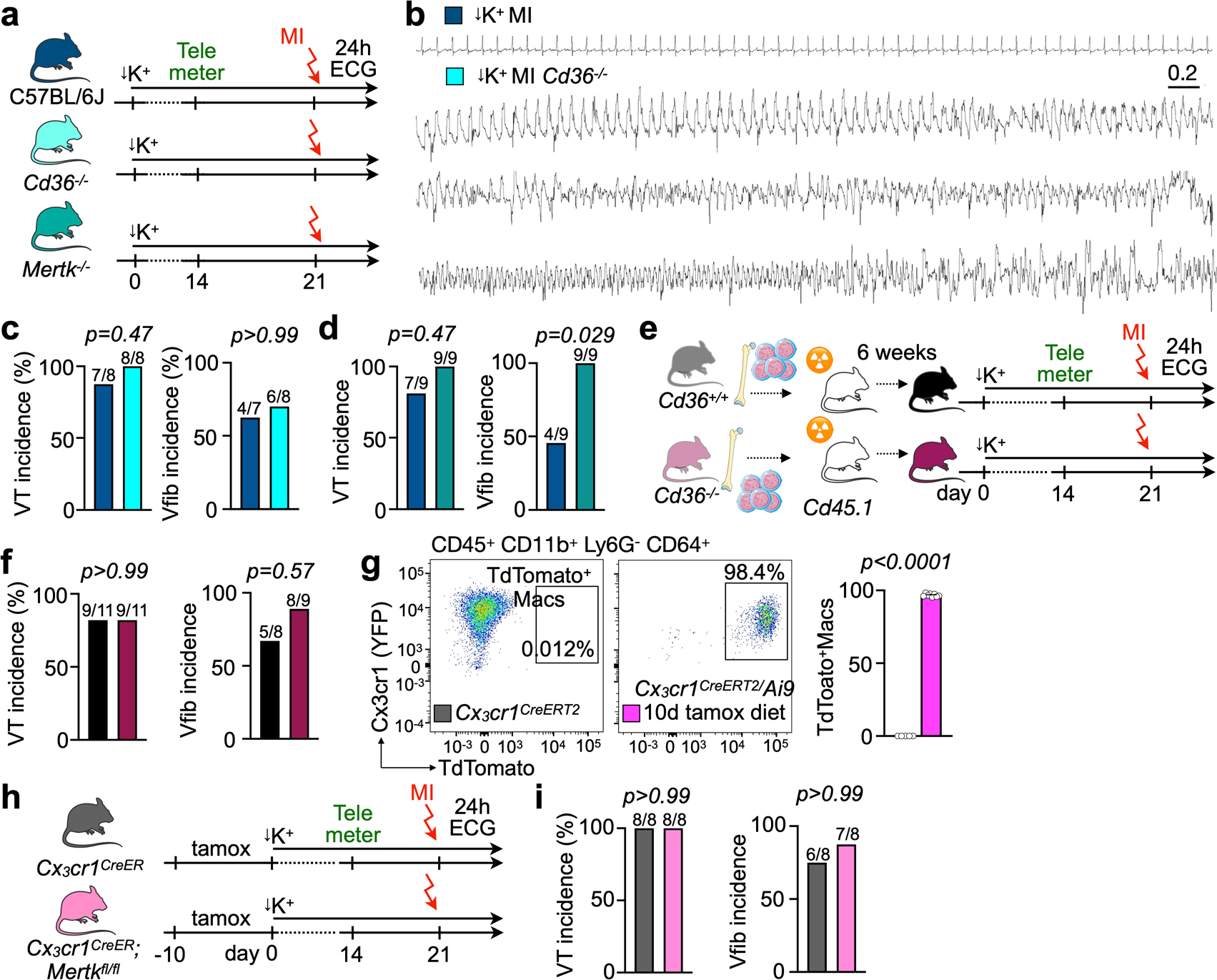
Scavenger receptor deletion facilitates post-MI ventricular. **a**, Experimental outline for C57BL/6 controls, *Cd36*^*−/−*^ and *Mertk*^*−/−*^ mice, all undergoing STORM. **b**, ECG tracing from a *Cd36*^*−/−*^ mouse experiencing sustained ventricular tachycardia (VT) transforming into ventricular fibrillation (Vfib) and leading to sudden cardiac death shortly after MI induction. **c**, VT incidence and Vfib incidence (mouse numbers in plots) from telemetric ECG recordings in ambulatory C57BL/6 STORM mice (n = 7 mice) and *Cd36*^*−/−*^ STORM mice (n = 8). Two-sided Fisher’s exact tests were used. **d**, VT incidence and Vfib incidence (mouse numbers in plots) from telemetric ECG recordings in ambulatory C57BL/6 STORM mice (n = 9 mice) and *Mertk*^*−/−*^ STORM mice (n = 9). Two-sided Fisher’s exact tests were used. **e**, Experimental outline for bone marrow transplantation using wild type *Cd36*^*+/+*^ or *Cd36*^*−/−*^ donor mice. Lethally irradiated recipients were *Cd36*^*+/+*^ mice that subsequently underwent STORM. **f**, VT incidence and Vfib incidence (mouse numbers in plots) from telemetric ECG recordings in ambulatory *Cd36*^*+/+*^ control bone marrow chimeras (n = 8 mice) and *Cd36*^*−/−*^ bone marrow chimeras (n = 9) after STORM procedure. Two-sided Fisher’s exact tests were used. **g**, Flow plots demonstrating Cre activity in resident cardiac macrophages of Cx_3_cr1^CreERT2/^Ai9 mice after consuming tamoxifen diet for 10 days. Unpaired t-test was used. **h**, Experimental outline. *Cx*_*3*_*cr1*^*CreER*^;*Mertk*^*fl/fl*^ mice and *Cx*_*3*_*cr1*^*CreER*^ control mice were fed with tamoxifen (tamox)-containing diet for 10 days. **i**, VT incidence and Vfib incidence (mouse numbers in plots) from telemetric ECG recordings in ambulatory *Cx*_*3*_*cr1*^*CreER*^ controls and *Cx*_*3*_*cr1*^*CreER*^;*Mertk*^*fl/fl*^ mice after STORM procedure. Two-sided Fisher’s exact tests were used. Data are mean ± SEM.

**Fig. 7 |:**
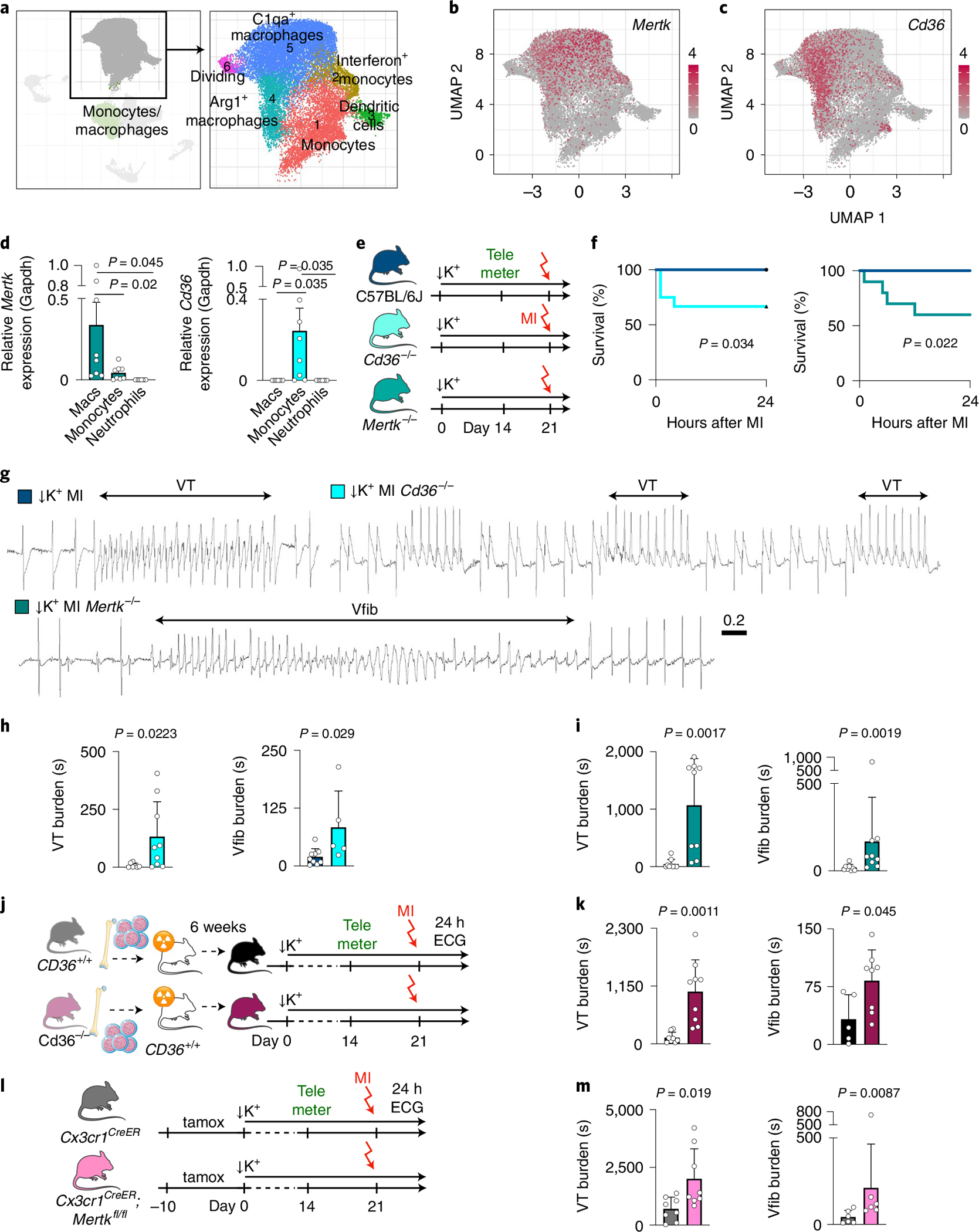
Phagocytosis receptor deletion causes sudden cardiac death. **a**, scRNA-seq data from *n* = 3 mice each on days 1, 2 and 4 after MI. Uniform manifold approximation and projection (UMAP) indicates cell subset. **b**, UMAP of *Mertk* expression by cell populations shown in **a**. **c**, UMAP of *Cd36* expression by cell populations shown in **a**. **d**, *Mertk* and *Cd36* expression in flow-sorted macrophages (Macs) (*n* = 8 mice), monocytes (*n* = 8 mice) and neutrophils (*n* = 7 mice) 5 hours after MI, normalized to *Gapdh*. One-way ANOVA followed by Tukey’s multiple comparisons test were used for statistical analysis. **e**, Experimental outline. C57BL/6, *Cd36*^*−/−*^ and *Mertk*^*−/−*^ mice underwent STORM protocol. **f**, Kaplan–Meier survival curve of wild-type (*n* = 14 mice, left, and *n* = 11 mice, right), *Cd36*^*−/−*^ (*n* = 14) and *Mertk*^*−/−*^ (*n* = 10) mice. *P* values were calculated using the log-rank (Mantel–Cox) test. **g**, ECG recordings from C57BL/6, *Cd36*^*−/−*^ and *Mertk*^*−/−*^ mice, all after STORM procedure. **h**, VT burden and Vfib burden in C57BL/6 mice (*n* = 9) and *Cd36*^*−/−*^ mice (*n* = 9) after STORM procedure, within 6 hours after MI. A two-sided Mann–Whitney test was used for statistical analysis. **i**, VT burden and Vfib burden in C57BL/6 mice (*n* = 8) and *Mertk*^*−/−*^ mice (*n* = 9) after STORM procedure, within 6 hours after MI. Two-sided Mann–Whitney tests were used for statistical analysis. **j**, Experimental outline. Bone marrow donors were either wild-type or *Cd36*^*−/−*^ mice. Recipient wild-type mice underwent STORM protocol. **k**, VT burden and Vfib burden in wild-type controls (*Cd36*^*+/+*^, *n* = 8 mice) or *Cd36*^*−/−*^ (*n* = 9) bone marrow chimeras 24 hours after MI. Two-sided Mann–Whitney tests were used for statistical analysis. **l**, Experimental outline. *Cx3cr1*^*CreERt*2^;*Mertk*^*fl/fl*^ mice and *Cx3cr1*^*CreERt*2^ control mice were fed a tamoxifen (tamox) diet for 10 days. All mice underwent STORM protocol after tamoxifen exposure. **m**, VT burden and Vfib burden in *Cx3cr1*^*CreERt*2^ controls (*n* = 8 mice) or *Cx3cr1*^*CreERt*2^;*Mertk*^*fl/fl*^ mice (*n* = 8) 24 hours after MI. A two-sided unpaired *t*-test (VT burden) and a Mann–Whitney test (Vfib burden) were used for statistical analysis. Data are mean ± s.e.m.

Macrophage scavenger receptors recognize phosphatidylserine on apoptotic cells, thus enabling efferocytosis^40^. Cluster of differentiation (Cd) 36 facilitates apoptotic and necrotic cardiomyocyte uptake, particularly during the early hours after MI^41^. Cd36-enabled efferocytosis permits cardiac repair after ischemia^32^. The MER receptor tyrosine kinase (Mertk) similarly mediates efferocytosis and, in addition, facilitates the removal of dysfunctional mitochondria from cardiomyocytes^10^. To test the role of these receptors in MI-related ventricular arrhythmia, we first examined their expression by monocytes and macrophages after MI. In available scRNA-seq data obtained on days 1–4 after MI^21^ (Fig. 7a–c), followed up with quantitative RT–PCR in cells flow sorted 5 hours after MI (Fig. 7d), we found *Mertk* predominantly expressed by macrophages. *Cd36* was highly expressed by recruited monocytes at 5 hours after MI, but it was more broadly expressed by macrophages in the scRNA-seq data that were acquired at later timepoints when monocytes may have differentiated (Fig. 7c,d). Neither gene was expressed by neutrophils (Fig. 7d). We then exposed *Cd36*^*−/−*^ and *Mertk*^*−/−*^ mice to the STORM procedure. Unlike wild-type STORM controls, *Cd36*^*−/−*^ and *Mertk*^*−/−*^ STORM mice developed sudden cardiac death, as long VT episodes deteriorated into Vfib (Fig. 7e–g and Extended Data Fig. 7a,b). Of note, we did not observe myocardial rupture, which typically happens several days after an infarct and, therefore, likely did not contribute to the high mortality recorded here. Both *Cd36*^*−/−*^ and *Mertk*^*−/−*^ mice had increased VT and Vfib burden (Fig. 7h,i). Interestingly, *Mertk*^*−/−*^ STORM mice, but not *Cd36*^*−/−*^ STORM mice, had a higher Vfib incidence than STORM controls (Extended Data Fig. 7c,d), perhaps due to differences in cell subset expression of phagocytosis receptors. We next used chimeras, in which only bone-marrowderived leukocytes lacked *Cd36* expression, to specifically exclude contributions of Cd36 expressed by cardiomyocytes. Wild-type controls were also lethally irradiated but received *Cd36*^*+/+*^ bone marrow. When these cohorts underwent the STORM protocol, *Cd36*^*−/−*^ chimeras had elevated VT and Vfib burden, confirming the key leukocyte contribution to the anti-arrhythmic function of Cd36 (Fig. 7j,k and Extended Data Fig. 7e,f). Finally, we bred *Cx3cr1*^*CreERt2*^ mice, which efficiently target macrophages upon tamoxifen exposure (Extended Data Fig. 7g), with *Mertk*^*fl/fl*^ mice, resulting in macrophage-specific *Mertk* deletion. When exposed to the STORM procedure, *Cx3cr1*^*CreERt2*^;*Mertk*^*fl/fl*^ mice presented with increased VT and Vfib burden (Fig. 7l,m and Extended Data Fig. 7h,i), similarly to mice with global *Mertk* deletion. These data confirm that macrophage expression of *Mertk* protects against post-MI ventricular arrhythmias.

## Discussion

**Fig. 8 |:**
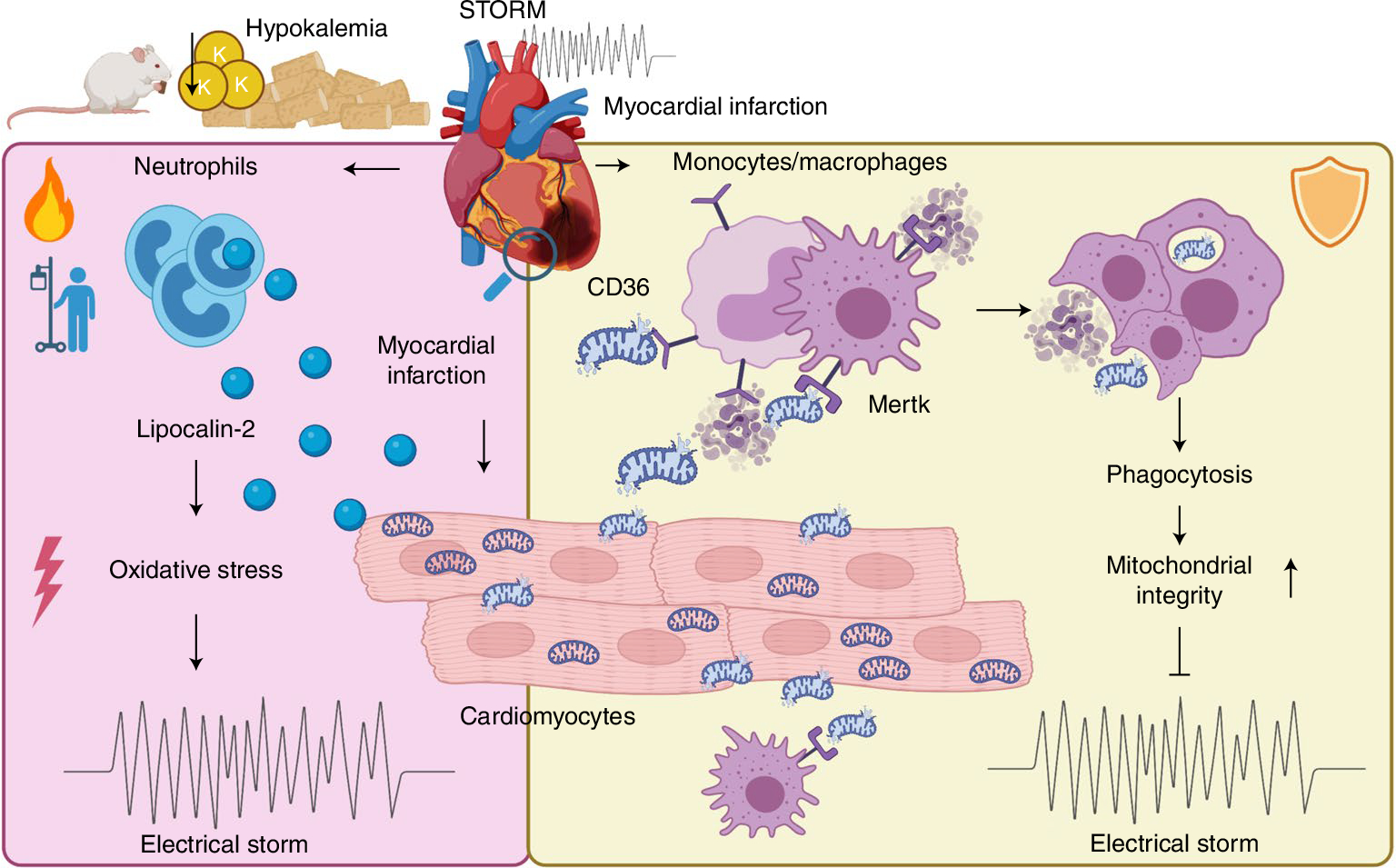
Summary of findings.

Myocytes and the specialized conduction system cells are responsible for cardiac excitation, and these cells’ dysfunction is the primary cause of arrhythmias. It has been known for decades that stromal cells may affect the cardiac rhythm by interacting with conducting cells. For instance, fibroblasts influence conduction indirectly via matrix deposition and directly via electrotonic coupling^42^. That macrophages participate in conduction is a more recent insight^9^, as even the existence of resident cardiac macrophages has emerged only recently^8,43^. Even though it remains unsettled how exactly leukocytes participate in arrhythmogenesis^44^, it is generally accepted that inflammation propagates rhythm disorders^45^. This notion rests on the clinical association of arrhythmia with inflammatory disorders—for example, myocarditis or sepsis—and with blood biomarkers, such as C-reactive protein or IL-6 (ref. ^44^). Furthermore, genetically enforced inflammasome activation in cardiomyocytes leads to inducible atrial arrhythmia in mice^46^. In the current work, we have identified how the most abundant cardiac leukocyte populations, specifically neutrophils and macrophages, influence ischemia-induced ventricular arrhythmias (Fig. 8).

Neutrophil depletion reduced VT burden, identifying these cells as proponents of ventricular arrhythmia in mice with an acute MI. A key neutrophil defense protein, Lcn2, increases ROS in cardiomyocytes^24^. Lcn2 could, thus, modulate ion channel proteins and their function by oxidation, which might change action potential duration and calcium handling. These alterations induce heterogeneity in conduction velocity, delayed afterdepolarizations and re-entry that underlie VT and Vfib^20^. We found that, similarly to neutrophil depletion, deleting Lcn2 from bone-marrow-derived leukocytes reduced the arrhythmia burden. These data align well with the observation that the prototypical neutrophil enzyme myeloperoxidase promotes atrial fibrillation via oxidative protein modifications^47^.

In contrast to neutrophils, macrophages protect against post-MI arrhythmias. Either inhibiting the Csf1 receptor, which reduces all macrophages irrespective of their source, or genetically deleting the chemokine receptor Ccr2, which abrogates recruitment of a macrophage subset considered inflammatory^48^, increased VT burden. A primary function of macrophages early after MI is phagocytosis of dead cardiomyocytes, a process that promotes wound healing after ischemia^11^. Removal of dead cells by monocytes and macrophages relies on Cd36 and Mertk receptors^31,32,41^. Genetic deletion of these receptors led to lethal arrhythmias in mice with acute MI. Impaired removal of dead or damaged cells may slow regional conduction and increase electrical heterogeneity in the myocardium, both of which are potential substrates for re-entry and ventricular arrhythmias^6,49^. In addition, our data suggest that macrophages may decelerate myocyte death during ischemia. Although infarct size 24 hours after permanent coronary ligation, which is governed by the site of the coronary artery ligation, was similar in mice with and without macrophages, TUNEL and caspase assays obtained 5 hours after MI, when VT and Vfib were most common, indicated that myocytes perished more slowly if macrophages were present. Because cardiac resident macrophages preserve myocytes’ metabolic health through scavenging dysfunctional mitochondria^10^, it is conceivable that the lack of macrophages, which preceded ischemia in our experiments, hastened mitochondrial failure. Indeed, depleting macrophages accelerated mitochondrial membrane potential collapse in ischemic myocytes. This, in turn, may have drained ATP faster, possibly jeopardizing ion pump function and Ca handling^50^. Additional mechanisms influencing cardiomyocyte autophagy^51^ might contribute to the accumulation of dysfunctional mitochondria in the absence of macrophages. Ultimately, mitochondrial failure induces cell death^34^, a catastrophic event that produces complete loss of regional conduction. The resulting local block may add to the myocardium’s electrical heterogeneity.

The observed protective macrophage functions after ischemia accord with their supportive roles in AV node conduction^9,52^. These cells facilitate conduction via gap junction coupling^9^ or, in the setting of pulmonary hypertension, by secreting amphiregulin, which preserves gap junction communication between myocytes^52^. Macrophage death during ischemia may deprive the ischemic myocardium of such support. We suspect that there are likely additional aspects to macrophages’ beneficial roles, such as modulating sympathetic cardiac innervation^53^, cytokine signaling or scavenging tissue microenvironment, which may, in turn, influence the ability of myocytes to survive or conduct.

We began investigating the causal roles of leukocytes in ventricular arrhythmia by studying mice with no comorbidities beyond ischemia and hypokalemia. Although this is a reasonable starting point, it differs from the clinical scenario, which includes comorbidities and cardiovascular risk factors. Such comorbidities may raise systemic leukocyte numbers and skew the cell repertoire toward inflammatory phenotypes^54^. We found that depleting macrophages or their receptors promoted post-MI electrical storm; however, macrophage oversupply may also be pro-arrhythmic. In *apoE*^*−/−*^ mice with pre-existing atherosclerosis, rapid pacing more efficiently induces ventricular arrhythmia on day 5 after ischemia^55^. In mice and patients, atherosclerosis increases leukocyte production, which, in turn, elevates infarct inflammation^26^. Thus, future studies should evaluate how macrophage oversupply, or inflammatory phenotypic bias, influences arrhythmia. Our clinical data on the pro-arrhythmic effects of neutrophils support such a hypothesis. The contribution of cardiac leukocytes to arrhythmia likely varies according to the underlying substrate, presumably with a lower contribution to VT arising from chronic scarring and greatest relevance for conditions with acute inflammatory myocardial injury, including infarction as tested here and, potentially, also myocarditis, cardiomyopathies or sarcoidosis.

These follow-up questions—for example, regarding the roles of leukocytes during reperfusion or how to identify harmful macrophage and lymphocyte subsets—can be addressed with the straightforward STORM mouse model of spontaneous VT and Vfib. Because STORM does not rely on genetic manipulation of cardiomyocytes, it enables leukocyte-specific and fibroblast-specific genetic loss-of-function studies. The STORM model also has limitations, most of which it shares with any arrhythmia studies in mice. Compared to humans and large animals, mice have a higher heart rate, a shorter and differently shaped action potential, diverging ion channel functions and a smaller ventricle that may affect re-entry^12^. A specific limitation of the STORM model pertains to its reliance on hypokalemia, as only a subset of patients is hypokalemic. It is, therefore, important to examine leukocyte actions on ventricular arrhythmia in more suitable large animals, such as normokalemic pigs or dogs^56^. Ultimately, only prospective clinical trials will illuminate whether leukocyte-targeted interventions reduce arrhythmia in humans.

This work was directed by clinical questions and shows that neutrophil depletion could be a therapeutic opportunity to curb ischemia-triggered electrical storm. However, beneficial properties of neutrophil subsets after MI^57^ indicate that broad cell depletion may be problematic. Co-existing beneficial and detrimental cell functions are a common phenomenon for immune cells^58^. For example, in the setting of bacterial meningitis, neutrophils defend against infection by removing bacteria but also inflict permanent nerve damage that can lead to deafness in the survivors^59,60^. Neutralizing specific pro-arrhythmic neutrophil products, perhaps even lipocalin-2, may limit negative side effects on infarct healing and immune defense. To our surprise, all macrophage subsets, including monocyte-derived macrophages that often fuel detrimental inflammation, appear to protect against post-MI arrhythmia, raising the possibility that overzealous macrophage targeting enables arrhythmia. Csf1R and CCR2 inhibition, or other immunotherapeutics that interfere with the heart’s leukocyte reservoir, may compromise cardiac mitochondrial health, myocyte metabolism and conduction. Taken together, our results demonstrate that leukocytes are causally implicated in ischemia-induced VT, which motivates studying participation of this cell class in electrophysiological pathologies beyond MI, especially in conditions with inflammatory components. Understanding specific arrhythmia-promoting immune cell functions may enable developing a new class of immunomodulatory anti-arrhythmic drugs.

## Methods

### Human subjects.

Patients with MI (STEMI) who underwent PPCI at Oxford University Hospitals were enrolled into the Oxford Acute Myocardial Infarction study between 2010 and 2020 (ref. ^61^), which complies with the Declaration of Helsinki and was approved by a local research ethics committee (10/H0408/24). Patients were excluded if they had a late presentation with symptom duration >12 hours, cardiogenic shock, previous coronary artery bypass grafting, severe heart valve disease, contraindication to MRI, age >85 years, diagnosis of sepsis or infection during the same episode or use of immunosuppressants. Verbal assent at the time of PPCI was followed by informed written consent. No financial compensation was offered for participation, although study-related travel expenses could be reimbursed. After PPCI, patients underwent continuous ECG monitoring for at least 24 hours^62^. These records were used to establish a prospectively defined arrhythmia score for each patient: (i) no arrhythmia, (ii) ventricular ectopic beats, (iii) non-sustained VT and (iv) sustained VT (>30 seconds or requiring cardioversion) or Vfib. Patients underwent MRI at a median of 2 days after PPCI^61,63^. Statistical analyses for the Oxford cohort were performed using GraphPad Prism 9 (GraphPad Software), IBM SPSS for Macintosh version 28.0.0.0 (version 27.0, IBM) and RStudio version 1.4.1717. MRI analyses were conducted using cvi42 (Circle Cardiovascular Imaging). Statistical testing for the presence of a trend across arrhythmia score groups according to the neutrophil count was performed using the Jonkheere–Terpstra test^64^ as implemented in the clinfun package^65^ in R version 4.1.1. Clinical risk factors for arrhythmias include cardiogenic shock, late presentation after onset of symptoms and larger infarct size while prior beta-blocker usage is protective^62,66^. Patients with cardiogenic shock or presentation >12 hours from onset of symptoms were excluded. To adjust for ischemic time, beta-blockers and the MI size measured using MRI, logistic regression analysis was performed. The first level of the binary outcome was the composite of no arrhythmia or ventricular ectopic beats, and the second level of the binary outcome was the composite of non-sustained VT, sustained VT or Vfib. The logistic regression model was statistically significant (*P* < 0.001), correctly classifying 68% of cases. Neutrophil count (*P* < 0.001) and acute MI size (*P* = 0.004) contributed significantly to the model, whereas the use of beta-blockers (*P* = 0.53) and ischemic time (*P* = 0.57) did not. Neutrophil count continued to contribute significantly to the adjusted model (each 1 × 10^9^ per L increase in neutrophil count was associated with a 20% relative increase in the risk of ventricular arrhythmia (HR = 1.20, 95% CI: 1.056–1.36, *P* = 0.003)) as did MI size (for each 1% increase in acute MI size, HR = 1.02, 95% CI: 1.000–1.048, *P* = 0.048).

To investigate links between neutrophil counts and MI outcomes, human subject data were collected retrospectively at Mass General Brigham. The research was approved by the Partners Healthcare Institutional Review Board, and the need for individual informed consent was waived. No financial compensation was offered. The Partners Data Warehouse is linked to the Social Security Death Index, assuring a high degree of completeness of follow-up. We collected data from hematological testing and clinical MI outcomes between June 2015 and June 2020. Inclusion criteria included (1) diagnosis of NSTEMI or STEMI, (2) increased troponin, (3) white cell differential within 24 hours before or 48 hours after the first positive troponin and (4) age 40–85 years. Patients were excluded if they had sepsis or infection during MI. For patients with multiple neutrophil counts, the maximum value was used. Analyses for the Mass General Brigham cohort were performed using GraphPad Prism 9, IBM SPSS for Macintosh version 28.0.0.0 (version 27.0) and RStudio version 1.4.1717, implementing the survival package (version 3.2–13) downloaded from https://CRAN.R-project.org/package=survival. The pre-specified clinical outcomes of interest were a composite of cardiac arrest or all-cause death at 30 days. The cohort was dichotomized at the median neutrophil count of 6.6 × 10^9^ per L. We used the proportional hazard test^67^ as implemented by the cox.zph command in the Survival package^68^ in R version 4.1.1 to test the proportional hazards assumption against non-proportional hazards (that is, time-varying coefficients). For time-to-event analysis, the start date was defined as the date of the positive troponin test triggering a diagnosis of MI. To adjust for covariates, a series of Cox regression models adjusted for the effects of age; sex; peak troponin level; STEMI diagnosis; other components of the white cell count differential, including peak monocyte count and peak basophil count; and peak creatinine were constructed.

### Mice.

Experiments were approved by the Massachusetts General Hospital Institutional Animal Care and Use Committee, performed in compliance with relevant ethical regulations, and all efforts were made to avoid suffering of animals. Wild-type C57BL/6J, tamoxifen-inducible *B6J.B6N(Cg)-Cx3cr1tm1.1(cre)Jung/J* (*Cx3cr1*^*CreERT2*^), *B6.SJL-Ptprc*^*a*^ *Pepc*^*b*^*/BoyJ* (*Cd45.1*), *B6.129S4-Ccr2tm1Ifc/J* (*Ccr2*^*−/−*^), B6.129P2-*Lcn2tm1Aade*/*AkiJ* (*Lcn2*^*−/−*^), *B6.129S1-Cd36tm1Mfe/J* (*Cd36*^*−/−*^), *B6;129-Mertktm1Grl/J* (*Mertk*^*−/−*^), B6.129(Cg)-Gt(ROSA)26Sor^tm4(ACTB-tdTomato,-EGFP) Luo/J^, B6.FVB-Tg(Myh6-cre)2182Mds/J and *B6.Cg-Gt(ROSA)26Sortm9(CAG-tdTomato)Hze/J* (*Ai9*) mice were purchased from The Jackson Laboratory. *Mertk*^*fl/fl*^ mice were provided by Carla Rothlin (Yale University)^49^. *B6.Cg-Tg(Myh6-GCaMP8)B4–10Mik/J* (*Myh6-GCaMP8*) mice were provided by Cornell Heart Lung Blood Resource for Optogenetic Mouse Signaling. *Ly6G-Cre* mice were provided by Mikael Pittet (Massachusetts General Hospital; breeders received from the Institute for Experimental Immunology and Imaging, University Hospital Essen) and were bred to *Ai9* mice to generate *Ly6G*^*Tdtomato*^ mice, as reported previously^47^. Experiments were performed in 8–20-week-old, age-matched male and female mice. Housing conditions followed a 12-hour dark/light cycle, room temperature of 18–22 °C and maintained humidity between 40% and 60%. Mice were fed with respective diets ad libitum. Wild-type controls were C57BL/6J, as recommended by the vendor, for studies in *Ccr2*^*−/−*^, *CD36*^*−/−*^ and *Mertk*^*−/−*^ mice. To establish hypokalemia, mice consumed a potassium-deficient diet (background potassium: 15–30 p.p.m., TD.88239, Envigo) for 3 weeks. Macrophages were depleted by feeding mice a potassium-deficient diet containing Csf1R inhibitor PLX-5622 (1,200 p.p.m., MedChemExpress, and TD.200711, Envigo)^42^. Neutrophils were depleted by injections of anti-Ly6G (BE0075–1, Bio X Cell) and mouse IgG2a anti-rat antibody (BE0122, Bio X Cell, each 100 μg d^−1^, intraperitoneal (i.p.)) for 3 days^29^. Control mice were injected with IgG2a isotype control antibodies (BE0085 and BE0089, both Bio X Cell). For activation of the Cre/loxP system in hemizygous Cx_3_cr1^CreER^, mice received tamoxifen-containing diet for 10 days (500 mg kg^−1^ diet, Td.130857, Envigo). Cre-carrying littermate controls were subjected to the same tamoxifen regimen as inducible knockouts. Where appropriate, animals were randomly assigned to experimental groups.

### Telemeter implantation.

For ETA-F10 transmitter (DSI) implantation, mice received buprenorphine (0.05 mg kg^−1^ of body weight, i.p. injection) before the procedure. Mice were anesthetized by inhalation of 2% isoflurane. An abdominal incision allowed insertion of a sterile ETA-F10 telemetry device (DSI). The ECG leads were fixed in a modified lead II position. Buprenorphine treatment was continued twice daily for 3 days after implantation.

### MI.

MI was induced by permanent ligation of the left coronary artery. Mice were anesthetized with 2% isoflurane, intubated and mechanically ventilated. A left thoracotomy was performed at the fourth intercostal space. The coronary artery was ligated with a monofilament nylon 8–0 suture (Ethicon). Animals were given buprenorphine before and twice daily for 3 days after MI.

### Electrophysiological studies.

An octapolar catheter (EPR-800, Millar) was inserted into the right jugular vein and positioned in the right atrium and ventricle. Ventricular effective refractory periods were measured using electrical stimulation with overdrive pacing trains at 100 ms, followed by single extra-stimuli. Ventricular arrhythmia induction was performed with triple extra-stimuli and pacing at gradually faster rates to a pacing cycle length of 10 ms.

### Electrolyte measurements.

Whole blood was centrifuged at 800*g* for 10 minutes at room temperature, and the K^+^, Cl^−^ and Na^+^ plasma levels were measured using a DRI-CHEM 7000 analyzer (Heska). For pH and iCa^2+^ measurements, blood was analyzed with an i-STAT CG4+ Cartridge (Patterson Veterinary Supply) using an i-STAT 1 system (FUSO Pharmaceutical Industries).

### Bone marrow transplantation.

Mice were lethally irradiated with a 9.5-Gy single shot. One day later, 5 × 10^6^ bone marrow cells were transplanted via intravenous injection.

### Flow cytometry and cell sorting.

Heart tissue was minced and enzymatically digested using collagenase I (450 U ml^−1^), collagenase XI (60 U ml^−1^), DNAse and hyaluronidase (60 U ml^−1^) (Sigma-Aldrich). Single-cell suspensions were stained with CD45-PerCP/Cy5.5 (clone 30-F11, 1:600, 103132, BioLegend), CD64-APC (clone X54–5/7.1, 1:600, 139305, BioLegend), Ly6G-PE/Cy7 (clone 1A8, 1:600, 127617, BioLegend), Ly6C-FITC (clone HK1.4, 1:600, 128006, BioLegend), CD11b-BV510 (clone M1/70, 1:600, 101245, BioLegend) and DAPI (0.1%, F10347, Thermo Fisher Scientific). Blood samples from *Ly6G*^*Tdtomato*^ mice were stained with Ly6G-PE/Cy7 (clone 1A8, 1:600, 127617, BioLegend), CD115-BV605 (clone AFS98, 1:600, 135517, BioLegend), CD11b-APC (clone M1/70, 1:600, 101212, BioLegend), CD45-BV711 (30-F11, 1:600, 103147, BioLegend), CD3-APC/Cy7 (clone 17A2, 1:200, 100221, BioLegend), CD19-APC/Cy7 (clone 6D5, 1:300, 115529, BioLegend), B220-APC/Cy7 (clone RA3–6B2, 1:300, 103224, BioLegend), Nk1.1-APC/Cy7 (clone PK136, 1:300, 108724, BioLegend) and DAPI. Data were recorded on an LSRII flow cytometer with FACSDiva 6.1 and analyzed with FlowJo 10 software (BD Biosciences). For qRT–PCR measurements, cells were flow sorted on a FACSAria II (BD Biosciences) into 350 μl of lysis buffer (RNeasy Plus Micro Kit, Qiagen).

### Echocardiography.

Echocardiography was performed using a MX250s transducer (15–30 MHz, center transmit: 21 MHz, axial resolution: 75 μm) together with a Vevo 3100 Imaging System (FUJIFILM VisualSonics)^69^ during 2% isoflurane anesthesia.

### Confocal microscopy of the isolated heart.

*Myh6-GCaMP8* mice were retro-orbitally injected with anti-Ly6G-AF647 (clone 1A8, 127610, BioLegend) 30 minutes before MI induction. The excised heart was cannulated using an 18-gauge cannula and a 6–0 silk suture. After perfusion of the heart with pre-warmed KH buffer (NaHCO_3_: 25 mM, KH_2_PO_4_: 1.2 mM, C_6_H_12_O_6_: 11.1 mM, MgSO_4_: 1.2 mM, KCl: 4.7 mM, NaCl: 118 mM, CaCl_2_: 2.55 mM), a pacing wire was sutured to the left ventricular tissue for pacing at 8 Hz. To identify the infarct border zone, we used 5,000 blood flow determination beads (FluoSpheres Polystyrene Microspheres, F8891, Thermo Fisher Scientific). Data were acquired with a confocal microscope (FV1000-MPE, Olympus)^70^.

### FRI.

Mice were intravenously injected with Annexin-V750 (250 μl, PerkinElmer) 4 hours after MI, 1 hour before imaging. Image acquisition was done using an epifluorescence microscope (OV-110, Olympus) equipped with IV10-ASW 01.01.00.05 software with an image matrix of 512 × 512 and 0.0178 mm × 0.0178 mm pixels. Mice were intravenously injected with TMRE perchlorate (1.5 μl of a 10 mM stock solution diluted in PBS, MedChemExpress) 4.5 hours after MI induction. Carbonyl cyanide 4-(trifluoromethoxy) phenylhydrazone (FCCP, 5 μl diluted in 150 μl of PBS, MedChemExpress) was retro-orbitally injected 15 minutes before TMRE. Oxidative stress was imaged after intravenous injection of CellROX Deep Red Reagent (C10422, Thermo Fisher Scientific, 20 μl diluted in 100 μl of PBS). Hearts were sliced in 1-mm sections for immediate imaging using a Sapphire Biomolecular Imager (Azure Biosystems).

### Immunostaining.

Hearts were embedded in OCT (Thermo Fisher Scientific). For TUNEL, sections were stained with In Situ Cell Death Detection Kit, TMR red (12156792910, Sigma-Aldrich), anti-cardiac troponin I antibody (ab47003, Abcam 1:250) and a goat anti-rabbit IgG secondary antibody Alexa Fluor 488 conjugate (A-11034, Thermo Fisher Scientific, 1:100). Nuclei were counterstained with DAPI (D21490, Thermo Fisher Scientific, 1:3,000). Images were acquired with a digital slide scanner NanoZoomer 2.0RS (Hamamatsu). We harvested hearts from hypokalemic mTmG-Myh6^Cre^ reporter mice 5 hours after MI, and paraffin-embedded tissue sections were stained with Tom20 (D8T4N) rabbit monoclonal antibody (42406, Cell Signaling Technology, 1:50) for mitochondria, GFP antibody (ab13970, Abcam, 1:400) for cardiomyocytes and purified rat-anti-mouse CD107b (M3/84) (550292, BD Biosciences, 1:25, Mac-3) for macrophages, followed by goat anti-rabbit IgG Alexa Fluor 647 conjugate (A-21245, Thermo Fisher Scientific, 1:100), goat anti-chicken IgG Alexa Fluor 488 conjugate (A-11039, Thermo Fisher Scientific, 1:100) and goat anti-rat IgG Alexa Fluor 555 conjugate (A-21434, Thermo Fisher Scientific, 1:100). Images were acquired using an Olympus FV3000 system with a ×60 oil immersion imaging objective (UPLAPO60XOHR). To measure infarct size, slices were stained with a 2% 2,3,5-triphenyltetrazolium chloride solution (Sigma-Aldrich) and imaged with a flatbed scanner.

### TEM.

Specimens were excised from the infarct core and fixed in 2% paraformaldehyde/2.5% glutaraldehyde in 0.1 M cacodylate buffer, rinsed in 0.1 M cacodylate buffer and infiltrated in 1% osmium tetroxide. Samples were transferred into a 1:1 mix of propylene oxide and eponate resin. Then, 70-nm sections were cut using a Leica EM UC7 ultramicrotome, collected onto formvar-coated grids, stained with 2% uranyl acetate and Reynold’s lead citrate and examined in a JEOL JEM 1011 transmission electron microscope at 80 kV.

### RNA isolation and real-time PCR.

RNA was isolated with the RNeasy Mini Kit (74104, Qiagen) and the Plus Micro Kit (74034, Qiagen). High-Capacity RNA-to-cDNA Kit (4387406, Applied Biosystems) was used for reverse transcription. TaqMan gene expression assays were used with TaqMan Fast Universal PCR Master Mix (4366072, Applied Biosystems) and primers for Lcn2 (Mm01324470_m1), Casp3 (Mm01195085_m1), Mertk (Mm00434920_m1), Cd36 (Mm00432403_m1), Retnlg (Mm01346434_m1), Hdc (Mm00456104_m1), Fadd (Mm00438861_m1), Mlkl (Mm01244222_m1), Tradd (Mm01251029_m1) and Gapdh (Mm99999915_g1, VIC-MGB, Thermo Fisher Scientific). Samples were run on a 7500 Real-Time PCR System (Applied Biosystems), and target gene expression was normalized to Gapdh.

### Troponin ELISA.

Whole blood was centrifuged for 10 minutes at 800*g*, and the supernatant was analyzed using the Mouse Cardiac Troponin T ELISA Kit (MBS034636, MyBioSource).

### Enzyme activity assays.

For a Caspase-3 Assay Kit (ab39401, Abcam), infarct tissue was immersed in 400 μl of lysis buffer and disrupted using a homogenizer. To measure mitochondrial enzyme activities, we used the Complex II Enzyme Activity Microplate Assay Kit (ab109908, Abcam) and Complex IV Rodent Enzyme Activity Microplate Assay Kit (ab109911, Abcam).

### Telemetry analyses.

ECG recordings were analyzed using LabChart 8 (ADInstruments). VT were defined as (i) four or more consecutive broad QRS complexes of ventricular origin at a rate of >800 beats per minute, (ii) absence of the intrinsic QRS complex and (iii) dissociated or indistinguishable atrial activity^71^. Vfib was defined as disorganized electrical activity at a high rate, with pronounced variability in ECG waveform, peak–peak interval and height^71^. Incidence analysis included all mice in respective cohorts. For the analysis of VT and Vfib burden, we measured the cumulative time that mice spent in respective arrhythmia during the first 24 hours after MI. After clinical guidelines for atrial fibrillation^72–74^, as VT burden and Vfib burden are not commonly assessed, we included mice that had a VT or Vfib >0 seconds in the burden analyses. QTc was assessed in at least five cardiac cycles per mouse^75^.

### Analyses of EP studies.

Data from EP studies were analyzed using LabChart 8 Pro. All VTs <1 second were excluded.

### Echocardiography analyses.

Left ventricular volumes, ejection fraction and cardiac output were calculated from B-mode images using the semi-automated LVtrace-tool of Vevo LAB version 3.1.0 (FUJIFILM VisualSonics). All B-mode loops were traced twice to account for inter-beat variability. Peak velocities for E and A were assessed from pulsed-wave Doppler transmittal flow patterns and e′ from tissue Doppler profiles of five peaks.

### Analyses of confocal microscopy.

To identify regions of interest with Ca^2+^ hotspots, cine loops were cropped into series of 5–30 frames using ImageJ 1.8.0_172. A dyssynchrony map was generated by plotting the standard deviation (s.d.) of GFP indicating cytosolic Ca concentration over the length of the respective time series. This dyssynchrony map was used to define Ca^2+^ hotspots with the ImageJ plugin SparkMaster^76^ using a threshold of four times the s.d. The dyssynchrony map was merged with the AF-647 channel, and the distance from Ca^2+^ hotspots to neutrophils was quantified. Random spots were picked in each dyssynchrony map by using the Microsoft Excel function ‘randombetween’.

### Analyses of FRI.

Background mean fluorescent intensity (MFI) was determined by placing a region of interest (ROI) in the remote tissue with ImageJ 1.8.0_172. Annexin V-positive area was defined as MFI times 5 × s.d. of remote cardiac tissue. Annexin V-positive areas were thresholded, and the ROI was placed into the infarct area to determine target MFI. Background MFI and target MFI from individual cardiac slices were used to calculate the TBR. For analyses of mitochondrial membrane potential, background MFI was determined adjacent to tissue. Target ROIs were defined in the myocardium or in the infarct. Background MFI and target MFI from individual cardiac slices were used to calculate the TBR. Fluorescence intensity levels were displayed using a pseudo rainbow color scheme (OsiriX, Pixmeo SARL).

### RNA-seq analysis.

We analyzed deposited scRNA-seq data (GSE157244) consisting of immune cells isolated from infarcted murine hearts at day 1 (*n* = 3 mice; 8,687 cells), day 2 (*n* = 3 mice; 6,350 cells) and day 4 (*n* = 8 mice, 20,843 cells) after permanent left anterior descending ligation^33^. Analyses were performed with R package Seurat version 3. Count matrices were merged into a single unified dataset and filtered to remove cells with unique molecular identifier counts below 100. Normalization, scaling, variable feature selection and principal component analysis-based dimensional reduction were performed with default parameters. Clustering was performed using the shared nearest neighbor clustering algorithm with the Louvain method for modularity optimization, as implemented in the Seurat FindNeighbors and FindClusters functions. Differentially expressed genes between clusters were determined using a Wilcoxon rank-sum test.

### Histology data analyses.

To analyze TUNEL staining, ten FOVs per animal from the infarct were exported at ×40 using NanoZoomer NDP.view2 software (Hamamatsu). For each FOV, we manually counted troponin^+^ DAPI^+^ TUNEL^+^ cardiomyocytes using ImageJ 1.8.0_172. Infarct size was quantified using ImageJ 1.8.0_172.

### TEM data analyses.

Images of TEM samples were taken by a blinded observer using an AMT digital imaging system with proprietary image capture software (Advanced Microscopy Techniques). We used ImageJ 1.8.0_172 to quantify (i) mitochondria per FOV from images with ×8,000 magnification, (ii) mitochondrial area from FOVs with ×25,000 magnification, (iii) cristae and (iv) and paracrystalline inclusions from images with ×40,000 magnification. Free extracellular mitochondria were defined as mitochondria not separated by a cell membrane from the extracellular space.

### Statistical tests.

Statistical analyses were performed using GraphPad Prism 9. Results are reported as mean ± s.e.m. Normality was assessed using the Shapiro–Wilk normality test. For a two-group comparison, normally distributed datasets underwent an unpaired parametric two-tailed *t*-test, whereas non-normally distributed data were evaluated with a non-parametric two-tailed Mann–Whitney test. For multiple comparisons, we used an ANOVA followed by a Tukey’s post test or test for linear trend as appropriate. For a description of the statistical methods used for the human studies, see the relevant Methods section ‘Human subjects’.

### Reporting summary.

Further information on research design is available in the Nature Research Reporting Summary linked to this article.

### Data availability

The scRNA-seq data were previously deposited in the National Center of Biotechnology Information’s Gene Expression Omnibus and are accessible through accession number GSE157244 (K.R.K.). Additional data supporting the findings in this study are included in the main article and associated files. Source data are provided with this paper.

### Code availability

This manuscript does not report original code.

## Extended Data

**Extended Data Table 1 |:** Patient characteristics of Oxford cohort. Baseline characteristics of patients with MI with ST segment elevation (STEMI) and treated with PPCI entering OxAMI study between June 2012 and March 2020 and meeting study inclusion and exclusion criteria. Data are presented as median with interquartile range (IQR) or *n* (%).

Demographics	n=217 patients
Age (years)	60 [52, 68]
Male gender (n, %)	189 (87%)
**Coronary risk factors**	
Hypertension (n, %)	77 (35%)
Diabetes (n, %)	22 (10%)
Previous MI (n, %)	14 (6%)
Smoking history (n, %)	68 (31%)
Family history (n, %)	55 (25%)
**Pre-existing medications**	
Beta-blockers (n, %)	8 (4%)
ACE inhibitor/ARB (n, %)	15 (7%)
Statin (n, %)	15 (7%)
**Vital signs**	
Systolic BP (mmHg)	130 [113, 145]
Diastolic BP (mmHg)	78 [67, 88]
Heart rate (bpm)	74 [66, 87]
**Culprit coronary artery**	
LAD (n, %)	102 (47%)
LCx (n, %)	30 (14%)
RCA (n, %)	80 (37%)
Other (n, %)	4 (1%)
**PCI procedure**	
Ischemic time (minutes)	178 [122, 272]
Number of stents	1 [1, 1]
Stent length (mm)	25 [17, 32]
Stent diameter (mm)	3.5 [3, 4]
**Blood tests**	
White cell count (×10^9^ /L)	11.1 [8.9, 13.3]
Neutrophil count (×10^9^ /L)	8.3 [6.7, 10.7]
Monocyte count (×10^9^ /L)	0.9 [0.7, 1.2]
Peak troponin (ng/L)	42100 [9895, 50000]
Creatinine (mg/dL)	0.81 [0.70, 0.94]
**CMR scan (48 hours)**	
LV end diastolic volume (mL)	161 [132, 186]
LV ejection fraction (%)	48 [42, 53]
RV end diastolic volume (mL)	131 [107, 152]
RV ejection fraction (%)	57 [50, 62]
Area-at-risk (T1 mapping), %	39 [30, 51]
Infarct size (late gadolinium enhancement >5SD, %LV)	21 [12, 33]

**Extended Data Table 2 |:** Patient characteristics of Mass General Brigham cohort. Baseline characteristics of patients undergoing treatment for acute MI (including STEMI and NSTEMI) at Massachusetts General Hospital between June 2015 and June 2020 and meeting study inclusion and exclusion criteria. Data are presented as median and interquartile range (IQR) or *n* (%).

Demographics	n=795 patients
Age (years)	68 [60, 75]
Male gender (n, %)	528 (66%)
**Ethnicity**	
Black or African American	49 (6%)
Asian	33 (4%)
White or Caucasian	616 (77%)
Hispanic/Latino	3 (0.4%)
Other/unavailable	94 (12%)
**MI type**	
STEMI diagnosis	186 (23%)
NSTEMI diagnosis	609 (77%)
**Blood tests**	
Peak troponin (ng/L)	599 [150, 1935]
White cell count (×10^9^/L)	10.0 [7.4, 13.8]
Neutrophil count (×10^9^/L)	6.6 [4.7, 9.8]
Monocyte count (×10^9^/L)	0.8 [0.6, 1.1]
Basophil count (×10^9^/L)	0.04 [0.02, 0.05]
Creatinine (mg/dL)	1.2 [0.9, 1.8]

**Extended Data Table 3 |:** Cox models for Mass General Brigham cohort. Association between peak neutrophil count (dichotomized at the median) and the composite outcome of death or cardiac arrest at 30 days in patients with acute MI as determined by Cox proportional hazard models. An unadjusted and a series of adjusted analyses in which the relevant and available covariates were progressively incorporated were performed. The performance of each model was evaluated by calculating the Akaike information criterion (AIC) and Bayesian information criterion (BIC). Model 3 provided the lowest values for both, indicating better performance. The neutrophil count remained associated with the risk of 30-day death or cardiac arrest in all conditions trialed.

Groups dichotomized at the median neutrophil count					
Model	Group	HR (95% CI)	P value	AIC	BIC
Unadjusted	Neutrophils < 6.6×10^9^/L	Reference		950.3	953.2
	Neutrophils ≥ 6.6×10^9^/L	4.5 (2.5, 8.1)	<0.001		
Model 1	Neutrophils < 6.6×10^9^/L	Reference		941.0	949.5
	Neutrophils ≥ 6.6×10^9^/L	4.5 (2.5, 8.1)	<0.001		
Model 2	Neutrophils < 6.6×10^9^/L	Reference		937.8	952.0
	Neutrophils ≥ 6.6×10^9^/L	4.1 (2.3, 7.6)	<0.001		
Model 3	Neutrophils < 6.6×10^9^/L	Reference		722.5	745.2
	Neutrophils ≥ 6.6×10^9^/L	3.9 (1.9, 8.0)	<0.001		
					
**Model 1:** age, gender					
**Model 2:** age, gender, peak troponin, STEMI diagnosis					
**Model 3:** age, gender, peak troponin, STEMI diagnosis, peak monocyte count, peak basophil count, peak creatinine					

## Supplementary Material

Supplementary Video 1

Supplementary Video 2

Supplementary Table 1
