# Supplementary material for: Neutrophils incite and macrophages avert electrical storm after myocardial infarction
Source: Nat Cardiovasc Res. Author manuscript; Available in PMC 2022 Aug 25. (PMC9410341; doi:10.1038/s44161-022-00094-w)
Supplement: Supplementary Table 1 [file NIHMS1827933-supplement-Supplementary_Table_1.pdf]

Supplementary Table 1

Rank	Gene name	Full name	Fold change
1	Retnlg	resistin like gamma	4.11
2	S100a9	S100 calcium binding protein A9	4.03
3	Lcn2	lipocalin 2	3.92
4	S100a8	S100 calcium binding protein A8	3.73
5	Hdc	histidine decarboxylase	3.21
6	Acod1	aconitate decarboxylase 1	3.17
7	Ngp	neutrophilic granule protein	2.94
8	G0s2	G0/G1 switch gene 2	2.94
9	Ccl3	chemokine (C-C motif) ligand 3	2.86
10	Hcar2	hydroxycarboxylic acid receptor 2	2.83
11	Il1r2	interleukin 1 receptor, type II	2.77
12	Mxd1	MAX dimerization protein 1	2.64
13	Slfn4	schlafen 2	2.58
14	Egr1	early growth response 1	2.47
15	Cxcl2	chemokine (C-X-C motif) ligand 2	2.46
16	Fpr1	formyl peptide receptor 1	2.39
17	Gadd45a	growth arrest and DNA-damage-inducible 45 alpha	2.37
18	Samsn1	SAM domain, SH3 domain and nuclear localization signals, 1 Symbol;Acc:MGI:1914992]	2.37
19	Nlrp3	NLR family, pyrin domain containing 3	2.36
20	Ifitm1	interferon induced transmembrane protein 1	2.33
21	Cxcr2	chemokine (C-X-C motif) receptor 2	2.31
22	Wfdc21	WD repeat domain 31	2.28
23	Cd24a	CD24a antigen	2.27
24	Trem1	TNF receptor-associated factor 1	2.22
25	Ccrl2	chemokine (C-C motif) receptor-like 2	2.22
26	Il1rn	interleukin 1 receptor antagonist	2.18
27	Csf1	colony stimulating factor 1 (macrophage)	2.17
28	Pnrc1	proline-rich nuclear receptor coactivator 1	2.11
29	Fpr2	formyl peptide receptor 2	2.10
30	Clec4e	C-type lectin domain family 4, member e	2.10
31	Lmnb1	lamin B1	2.10
32	Chil1	chitinase-like 1	2.09
33	Nfkbiz	nuclear factor of kappa light polypeptide gene enhancer in B cells inhibitor, zeta	2.09
34	Slpi	sarcolemma associated protein, opposite strand transcript 2	2.07
35	Cxcl3	chemokine (C-X-C motif) ligand 3	2.04
36	Dmxl2	Dmx-like 2	2.04
37	Asprv1	aspartic peptidase, retroviral-like 1	2.02
38	Il1f9	interleukin 1 family, member 9	1.92
39	Cd300lf	CD300 molecule like family member F	1.88
40	Mmp8	matrix metallopeptidase 8	1.88
41	Il1b	interleukin 1 beta	1.88
42	Ets2	E26 avian leukemia oncogene 2, 3' domain	1.87
43	Tnfaip2	tumor necrosis factor	1.87
44	Tnfaip3	tumor necrosis factor, alpha-induced protein 2	1.85
45	Btg2	BTG anti-proliferation factor 2	1.84
46	Marcksl1	MARCKS-like 1	1.83
47	Il1a	interleukin 1 alpha	1.81
48	Clec4d	C-type lectin domain family 4, member d	1.81
49	Cd33	CD33 antigen	1.81
50	Srgn	steroid receptor RNA activator 1	1.73
